# Refractory angina: mechanisms and stratified treatment in obstructive and non-obstructive chronic myocardial ischaemic syndromes

**DOI:** 10.1093/eurheartj/ehaf284

**Published:** 2025-07-01

**Authors:** Ranil de Silva, Kevin Cheng, Timothy D Henry, Divaka Perera, Viviany R Taqueti, Ana Abreu, Fabienne Vervaat, Martha Gulati, Hiroaki Shimokawa, Felicita Andreotti, Juan-Carlos Kaski

**Affiliations:** National Heart and Lung Institute, Imperial College London, Brompton Campus, Level 2 Chelsea Wing, Sydney Street, London SW3 6NP, UK; Specialist Angina Service, Royal Brompton and Harefield Hospitals part of Guy’s and St Thomas’ NHS Foundation Trust, Level 2 Chelsea Wing, Sydney Street, London SW3 6NP, UK; National Heart and Lung Institute, Imperial College London, Brompton Campus, Level 2 Chelsea Wing, Sydney Street, London SW3 6NP, UK; Specialist Angina Service, Royal Brompton and Harefield Hospitals part of Guy’s and St Thomas’ NHS Foundation Trust, Level 2 Chelsea Wing, Sydney Street, London SW3 6NP, UK; The Carl and Edith Lindner Center of Research and Education, The Christ Hospital, 2123 Auburn Avenue, Suite 424, Cincinnati, OH 45219, USA; British Heart Foundation Centre of Research Excellence at the School of Cardiovascular and Metabolic Medicine & Sciences, King's College London, Westminster Bridge Road, London SE1 7EH, UK; Guy's and St Thomas’ NHS Foundation Trust, Westminster Bridge Road, London SE1 7EH, UK; Cardiovascular Imaging Program, Departments of Radiology and Medicine, Brigham and Women's Hospital, Harvard Medical School, 75 Francis Street, Boston, MA 02115, USA; Cardiovascular Rehabilitation Centre, Department of Cardiology, ULS Santa Maria and Faculdade Medicina de Universidade Lisboa (CAML), ISAMB, IMPSP, CCUL, Av. Professor Egas Moniz, 1649-028, Lisboa, Portugal; Department of Cardiology, Catharina Hospital, Michelangelolaan 2, Eindhoven 5623 EJ, the Netherlands; Barbra Streisand Women's Heart Center, Smidt Heart Institute, Cedars-Sinai Medical Center, 8700 Beverly Blvd., Los Angeles, CA 90048, USA; Graduate School, International University of Health and Welfare, Kozunomori 4-3, Narita 286-8686, Japan; Department of Cardiovascular Medicine, Tohoku University, Seiryo-machi 1-1, Sendai 980-8574, Japan; Cardiovascular Science Department, Fondazione Policlinico Universitario A. Gemelli IRCCS, Largo Gemelli 8, 00168 Rome, Italy; CardioThoracic Department, Catholic University Medical School, Rome, Italy; Molecular and Clinical Sciences Research Institute, St. George's University of London, Cranmer Terrace, London SW17 0RE, UK

**Keywords:** Refractory angina pectoris, Chronic coronary syndromes, INOCA, ANOCA, Non-acute myocardial ischaemic syndromes

## Abstract

The diagnosis of refractory angina has conventionally been limited to patients with angina and ischaemia secondary to obstructive atherosclerotic epicardial coronary disease who experience persistent symptoms despite optimal pharmacological and revascularization therapies. It is now well-established that angina may also be caused by ischaemia resulting from coronary microcirculatory disorders, coronary vasospasm, and bridging in the absence of obstructive epicardial coronary disease or after “successful” revascularization. This increasingly prevalent and symptomatic group of patients, with both angina and demonstrable ischaemia, have been excluded from the conventional definition of refractory angina. In patients with obstructive epicardial coronary disease, disturbed microcirculatory and vasomotor function, amongst other ischaemic mechanisms, may account for continuing symptoms despite revascularization. Under-recognition of these mechanisms results in inadequate treatment and symptom persistence. In this review, a redefinition of refractory angina is proposed to include the full spectrum of patients experiencing persistent angina despite current maximal guideline-directed medical and revascularization therapies. Systematic approaches for comprehensive investigation are suggested to identify underlying mechanisms of ischaemia and stratify treatments accordingly. The complex needs of patients with refractory angina are likely best addressed by an inter-disciplinary Angina Heart Team with the aim of improving patient symptoms, quality of life, and clinical outcomes.

## Graphical Abstract

**Graphical Abstract:**
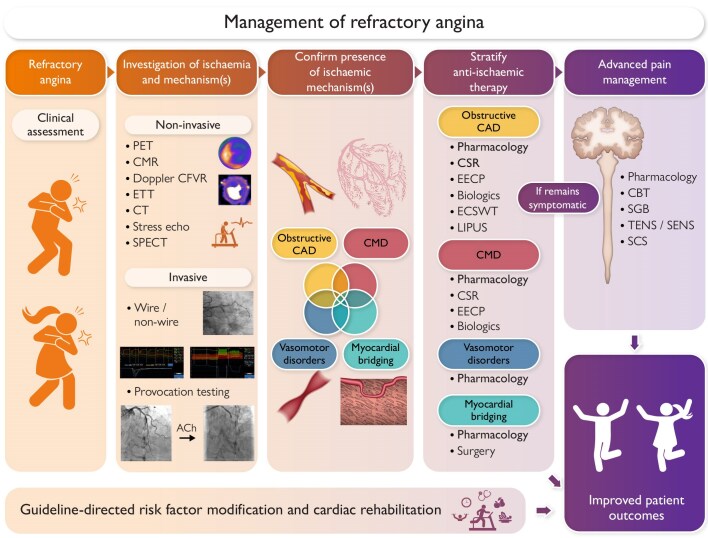
A contemporary approach to the stratified management of refractory angina resulting from obstructive and non-obstructive coronary artery disease. Guideline recommended stratified anti-ischaemic therapies are shown in bold. CAD, coronary artery disease; CBT, cognitive behavioural therapy; CFVR, coronary flow velocity reserve; CMD, coronary microvascular dysfunction; CMR, cardiac MRI; CSR, coronary sinus reducer; CT, computed tomography; ECSWT, extracorporeal shockwave therapy; EECP, enhanced external counterpulsation; ETT, exercise tolerance test; LIPUS, low-intensity pulsed ultrasound; PET, positron emission tomography; SCS, spinal cord stimulation; SENS, subcutaneous electrical nerve stimulation; SGB, stellate ganglion block; TENS, transcutaneous electrical nerve stimulation; VSA, vasospastic angina

## Introduction

Refractory angina is conventionally diagnosed in patients with angina and demonstrable ischaemia secondary to obstructive epicardial coronary artery disease (CAD) that persists despite maximally tolerated guideline-directed pharmacological therapy and achievable revascularization.^1^ It is well-established, however, that angina can also result from ischaemia triggered by other mechanisms, including coronary microvascular dysfunction (CMD), epicardial or microvascular vasospasm, and myocardial bridging. These can occur in the presence or absence of obstructive epicardial CAD, and in patients with persistent angina despite successful coronary revascularization.^2,3^ Patients without flow-limiting CAD who have demonstrable ischaemia due to CMD or vasospasm and experience life-limiting angina fall outside the current definition of refractory angina.^4^ Furthermore, CMD and vasospasm can additionally occur in patients with obstructive epicardial CAD, but frequently remain under-recognized and under-treated.^5^ Focusing exclusively on obstructive CAD may misclassify patients as having refractory angina, as symptoms might improve with appropriate stratified treatment. Thus, in patients deemed to have refractory angina, it is necessary to evaluate comprehensively the potential pathophysiological mechanisms causing myocardial ischaemia.

A re-definition of refractory angina is urgently needed to encompass the diversity of patients with angina encountered in contemporary clinical practice. In this article, approaches for the systematic non-invasive and invasive identification of ischaemic mechanisms arising from all compartments of the coronary circulation are proposed, emphasising the importance of quantitative methods. An approach to using results of these investigations to stratify treatment is suggested, understanding that multiple mechanisms may co-exist in an individual patient. An integrated approach combining established medical therapies for ischaemia, cardiac rehabilitation (CR), psychological therapies, and advanced pain management is proposed. Building on previous work, a more inclusive definition of refractory angina is suggested together with a model of care, delivered by a specialist inter-disciplinary Angina Heart Team, which may best address the complex needs of patients with refractory angina leading to improved symptoms, quality of life, and clinical outcomes.^3^

## Re-definition of refractory angina

Refractory angina is currently defined as ‘a chronic condition characterized by the presence of angina caused by coronary insufficiency in the presence of CAD, which cannot be controlled by a combination of medical therapy, angioplasty, and coronary bypass surgery. The presence of reversible myocardial ischaemia should be clinically established to be the cause of the symptoms. Chronic is defined as a duration of >3 months.’^1^ Patients with angina or angina-equivalent symptoms and ischaemia with no obstructive CAD (ANOCA/INOCA), or those with persistent symptoms despite successful revascularization, were not explicitly included, as indicated in previous reports.^6^ Moreover, the current definition may result in patients being inappropriately classified as having refractory angina because the mechanisms responsible for their symptoms are not identified and therefore not appropriately treated. For patients with obstructive CAD, a trial of optimized medical therapy stratified according to the underlying pathophysiological mechanisms of ischaemia should be attempted for 3–6 months before considering revascularization.^7^

Given the current limited definition of refractory angina, the following contemporary re- definition is proposed:

*Refractory angina pectoris is a chronic condition of at least 6 months duration caused by myocardial ischaemia triggered by obstructive CAD and/or other mechanisms, and is characterized by the persistence of angina or angina-equivalent symptoms despite maximally-tolerated stratified anti-ischaemic therapy and achievable indicated revascularization. Demonstration of myocardial ischaemia is required, including assessment of the epicardial coronary arteries, coronary microcirculation, coronary vasospasm, and other mechanisms known to trigger myocardial ischaemia. Ischaemia due to supply–demand mismatch is not limited to stress-induced ECG changes, myocardial perfusion defects, wall motion abnormalities, or flow-limiting CAD but can also include reduced coronary flow reserve (CFR) or other abnormalities on coronary function testing.*

## Epidemiology, healthcare burden, and management priorities

Precise epidemiology of the prevalence and incidence of refractory angina across the full pathophysiological spectrum of chronic coronary syndromes is lacking due to inconsistency and variability of the criteria used to define this patient cohort. As an approximation, published data on patients with persistent angina with or without obstructive epicardial CAD, including post-revascularization are considered.

### Refractory angina associated with obstructive epicardial CAD

In Europe, the annual incidence of refractory angina is estimated at 30,000–50,000 new cases annually.^1^ In the US, between 600,000 and 1.8 million patients have refractory angina, with ∼75,000 new cases diagnosed each year.^8^ As survival from CAD improves, combined with an aging population and that 7%–14% of patients undergoing angiography have anatomy unsuitable for revascularization,^3^ the incidence and prevalence of refractory angina due to obstructive CAD will increase.^9^ Persistent angina after percutaneous or surgical revascularization is common (see Supplementary data online, *Table S1*). In the ORBITA-2 trial, only 39.8% of patients randomized to contemporary PCI were angina-free at 12 weeks by Seattle Angina Questionnaire.^10^ Contemporary revascularization is associated with a rate of 20%–40% angina persistence. The reasons for this are explored and discussed further.

### Refractory angina in non-obstructive CAD

The American College of Cardiology (ACC) National Cardiovascular Data Registry indicates that 3–4 million patients have symptoms or myocardial ischaemia without obstructive epicardial CAD.^11^ A longitudinal study of patients with angina and non-obstructive CAD demonstrated worse outcomes in epicardial or microvascular (endothelial-dependent or independent) vasomotor dysfunction, compared to those without.^12^ Other studies have confirmed that patients with INOCA or with a low CFR have worse clinical outcomes,^13^ irrespective of sex or ethnicity.^14^ The prevalence and incidence of refractory angina in this population are presently unknown.

### Healthcare priorities in refractory angina

Observational studies of refractory angina patients with epicardial CAD highlighted improvement in symptoms and quality of life as key treatment priorities.^9,15^ Similar clinical needs characterize patients with non-obstructive CAD.^4^ Most refractory angina patients experience diagnostic delays with persistent symptoms significantly impacting their physical, mental, and social health. The need for greater awareness, improved diagnosis, and provision of evidence-based guidelines has been highlighted.^4^

Patients with recurrent angina and either obstructive or non-obstructive epicardial CAD experience high rates of hospitalization, undergo repeated investigations, and incur significant costs.^16–19^ In patients with refractory angina due to CAD, these have been estimated at ∼US$10,185 over 3 years.^20^ In those with non-obstructive CAD, average lifetime healthcare costs were $767,288.^18^ In the US, INOCA accrues an annual healthcare cost of $21 billion compared to $55 billion for obstructive CAD.^21^ Treatment strategies for refractory angina will require demonstration of reduced hospitalizations and healthcare costs.

## Mechanisms of myocardial ischaemia in patients with refractory angina

**Figure 1:**
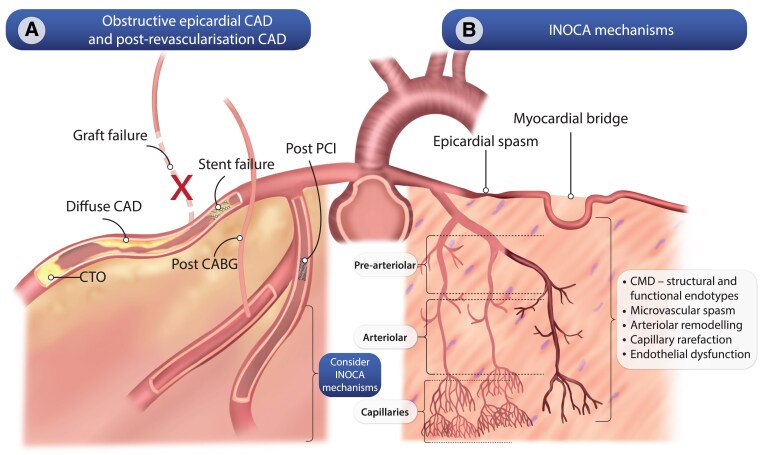
Summary of known mechanisms that cause ischaemia in patients with obstructive epicardial coronary artery disease with or without previous revascularization and ANOCA/INOCA at the level of both the epicardial coronary arteries and the coronary microcirculation. CABG, coronary artery bypass grafting; CAD, coronary artery disease; CTO, chronic total occlusion; INOCA, ischaemia and non-obstructed coronary arteries; PCI, percutaneous coronary intervention

Pathophysiological ischaemic mechanisms in refractory angina may originate from any compartment of the coronary circulation (*Figure 1*).^22^ Supply–demand mismatch may result from obstructive epicardial CAD after no attempted, failed, or initially successful revascularization. Moreover, mechanisms associated with INOCA such as vasospasm, myocardial bridging, and CMD may co-exist with CAD and with each other.^2,23–26^ Despite studies demonstrating an association between CMD and angina persistence after successful PCI, systematic investigation of ischaemic mechanisms other than obstructive epicardial CAD remains under-performed clinically.^27^ Treatment-resistant angina may simply result from a failure to consider and diagnose additional mechanisms. Therefore, a comprehensive approach combining clinical assessment and systematic investigation is suggested to identify ischaemic mechanisms and stratify therapy accordingly.

## Clinical assessment of patients considered to have refractory angina

A stepwise approach to patients with refractory angina is summarized in *Table 1*. A detailed clinical assessment including a careful history is essential to identify underlying ischaemic mechanisms and determine initial approach to investigation. Particular attention should be placed on symptoms and mechanisms associated with ANOCA/INOCA^28,29^ including coronary vasospasm.^30^ Variation in symptom reporting according to age, gender, and ethnicity should be considered.^29,31–34^ Conditions associated with anginal-type pain in addition to non-cardiac causes of chest pain should be considered (see Supplementary data online, *Table S2*).^35^ Trials of drugs aimed at improving suspected non-cardiac causes of chest pain, such as proton-pump inhibitors for gastro-oesophageal reflux, may be diagnostically and therapeutically useful.

**Table 1:** Algorithm for a step-by-step approach to the patient with refractory angina

	OBJECTIVES	ACTIONS
Step 1: clinical assessment	Confirm symptoms are angina Derive working hypothesis on underlying mechanism(s) of ischaemia to define investigational pathway	Review symptoms, risk factors, previous treatment, with attention on medication dosing and adherence Review previous anatomical and functional assessments Consider other conditions associated with anginal-type chest pain
Step 2: investigate for ischaemia	Confirm evidence of ischaemia as cause of symptoms Define underlying mechanism(s) of ischaemia	Non-invasive (ideally with quantitative modalities) Invasive (complete evaluation of epicardial CAD, muscle bridge, CMD, VSA)
Step 3: stratified treatment	Improve symptoms by addressing underlying mechanisms of ischaemia Confirm no revascularization options available Optimise risk factor management Improve quality of life and psychological wellbeing	Initiate and up-titrate appropriate pharmacologic and non-pharmacologic anti-ischaemic therapies stratified according to underlying mechanisms of ischaemia Treat to guideline-directed risk factor targets Consider referral to cardiac rehabilitation and cognitive behavioural therapy
Step 4: re-assess symptoms	Evaluate success of initial stratified treatment approach	Assess treatment response Modify and rationalise stratified treatment Assess treatment tolerability and adherence
Step 5: re-investigate if persistent symptoms (steps 2–4)	Review and refine working hypothesis	Consider multiple co-existing ischaemic mechanism(s) with appropriate modification of stratified therapy Reassess treatment tolerability and adherence Consider pain management strategies
Step 6: pain management for persistent symptoms	Improve symptoms and quality of life for symptoms which persist despite steps 2–5.	Refer for specialist pain management

CAD, coronary artery disease; CMD, coronary microvascular dysfunction; VSA, vasospastic angina.

## Invasive approach to ischaemia assessment

**Figure 2:**
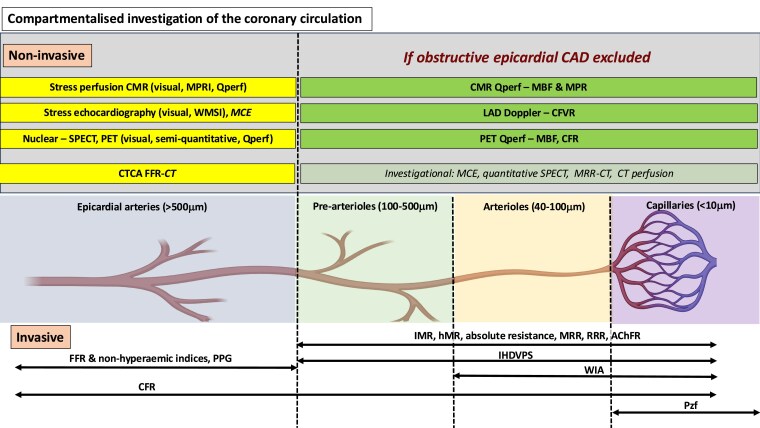
A compartmentalized approach to the non-invasive and invasive investigation of the coronary circulation to identify myocardial ischaemia. AChFR, acetylcholine flow reserve; CFR, coronary flow reserve; CFVR, coronary flow velocity reserve; CMR, cardiac magnetic resonance imaging; CT, computed tomography; CTCA, computed tomography coronary angiography; FFR, fractional flow reserve; FFR-CT, fractional flow reserve computed tomography; hMR, hyperaemic microvascular resistance; IHDVPS, instantaneous hyperaemic diastolic velocity-pressure slope index; IMR, index of microvascular resistance; LAD, left anterior descending artery; MBF, myocardial blood flow; MCE, myocardial contrast echocardiography; MRR, microvascular resistance reserve; MRR-CT, microvascular resistance reserve computed tomography; MPR, myocardial perfusion reserve; MPRI, myocardial perfusion reserve index; PET, positron emission tomography; Pzf, pressure at zero flow; PPG, pullback pressure gradient; Qperf, quantitative perfusion; RRR, resistance reserve ratio; SPECT, single-photon emission computed tomography; WIA, wave intensity analysis; WMSI, wall motion score index

In patients with refractory angina, invasive coronary angiography supplemented with haemodynamic and vasoreactivity measures is recommended.^36^ The specific methods for patients with obstructive and non-obstructive coronary arteries have been summarzed (*Tables 2 and 3* and *Figure 2*). A comprehensive approach to invasive evaluation is recommended to identify potential mechanisms of ischaemia in each compartment of the coronary circulation responsible for symptoms in an individual patient. Previous studies indicate that this is safe and easily incorporated into routine clinical workflow.^37–40^

**Table 2:** Invasive and non-invasive diagnostic approaches for obstructive epicardial coronary artery disease

Invasive assessments					
Modality	Metric	Cut-off	Clinical availability	Reproducibility	Reference
**Wire-based**					
Hyperaemic	FFR	≤0.80	Widely available	+++ Recommended in guidelines	[S1]
	CFR	<2.0	Widely available		[S2, 3]
	hSR	≥0.80 Hg/cm s	Currently not available	+++	[S4]
	PPG	0.73	Research	+++	[S5]
Non-hyperaemic	iFR RFR DFR Whole-cycle Pd/Pa	≤0.89 ≤0.91	Widely available	+++ Recommended in guidelines	[S6, 7]
				+++	
**Non-wire-based**					
Angiography-based	QFR	<0.80	Selected centres	+++ Recommended in guidelines	[S8–14]
	vFFR caFFR FFR_angio_			++	

Non-invasive assessments						
Modality	Stressor	Metric	Threshold	Clinical availability	Reproducibility	Reference
ECG	Exercise	ST-segment depression	≥0.1mV horizontal or down-sloping ST-segment depression 80msec from J point	Routine	+/++	[S15]
Cardiopulmonary exercise testing	Exercise	Peak VO_2_	ECG criteria as per exercise ECG + gas exchange analysis	Routine	++	[S16–21]
		O_2_ pulse flattening				
		ΔVO2/Δwork rate slope				
Computed tomography	N/A	Stenosis severity	70%	Routine	++	[S22]
		FFR-CT	≤0.80	Selected centres	+++	
		Perfusion	MBF index <78mL/100mL/min	Research or academic centre	Investigational	[S23]
**Stress Echocardiography**						
Wall motion assessment	Exercise Dobutamine Adenosine Dipyridamole	WMSI	New or worsening stress-induced segmental regional wall motion or thickening	Routine	++	[S24]
LAD Doppler	Adenosine Dipyridamole	CFVR	CFVR <2	Selected centres	+/++	[S25]
MCE perfusion	Adenosine Dipyridamole	Refill time	>2 s during vasodilator stress	Research or academic centre	Investigational	[S26]
**Nuclear**						
SPECT (^99m^Tc-sestabmibi, ^99m^Tc-tetrofosmin)	Exercise Adenosine Dipyridamole Regadenoson	Qualitative & summed scores	Qualitative or semiquantitative grading of reversible perfusion defect on rest/stress imaging	Routine	++	[S27]
Quantitative SPECT	Exercise Adenosine Dipyridamole Regadenoson	MBF MFR	Stress MBF 1.86mL/min/g MFR 1.61–1.95	Research or academic centre	+++	[S28, 29]
PET (^15^O, ^82^Rb, ^13^NH, ^18^F-flurpiridaz)	Adenosine Dipyridamole Regadenoson	CFR *CFC*	Qualitative or semiquantitative grading of reversible perfusion defect on rest/stress imaging CFR <2 *Graded severity thresholds*	Research or academic centre	+++	[S30]
**Perfusion CMR**						
Visual adjudicated	Adenosine Regadenoson	Visual perfusion defect	Segmental transmurality and persistence of stress perfusion defect compared with rest perfusion or late gadolinium enhancement	Routine	++	[S31]
Semi-quantitative	Adenosine, Regadenoson	MPRI	<1.1	Research or academic centre	++	[S32]
Quantitative	Adenosine, Regadenoson	MPR Stress MBF Endo:Epi ratio	Stress MBF <1.94	Research or academic centre	+++	[S33, 34]

CAD, coronary artery disease; caFFR, computation pressure-flow dynamics derived FFR; CFC, coronary flow capacity; CFR, coronary flow reserve; CFVR, coronary flow velocity reserve; CMR, cardiac MRI; DFR, diastolic hyperaemia-free ratio; ECG, electrocardiogram; endo:epi ratio, endocardial to epicardial ratio; FFR, fractional flow reserve; FFR_angio_, coronary angiography-derived FFR; FFR-CT; fractional flow reserve—computed tomography; hSR, hyperaemic stenosis resistance; iFR, instantaneous wave-free ratio; LAD, left anterior descending; MBF, myocardial blood flow; MCE, myocardial contrast echocardiography; MFR, myocardial flow reserve; MPR, myocardial perfusion reserve; MPRI, myocardial perfusion reserve index; MRI, magnetic resonance imaging; Pa, aortic pressure; Pd, distal coronary pressure; PET, positron emission tomography; PPG, pullback pressure gradient; QFR, quantitative flow ratio; RFR, resting full-cycle ratio; SPECT, single-photon emission computed tomography; VCO_2_, volume of carbon dioxide production; VE, minute ventilation; vFFR, vessel FFR; VO_2_, volume of oxygen uptake; WMSI, wall motion score index. Reproducibility: low (+), acceptable (++), good (+++). *Specific cut-off values for each modality are subject to ongoing investigation.*

**Table 3:** Invasive and non-invasive diagnostic approaches for evaluation of ischaemia not attributable to obstructive epicardial coronary artery disease (fully referenced in Supplementary Appendix)

Invasive assessments					
Modality	Metric	Cut-off	Clinical availability	Utility	Reference
**A. CMD assessment**					
**Wire-based**					
Intracoronary Doppler	CFR	<2.5	Currently unavailable	High and extensively validated Recommended in guidelines	[S35–37]
	hMR	>2.5			[S35, 36]
	Pzf	≥42mmHg			[S38]
	AChFR	≤1.5			[S39]
Bolus thermodilution	CFR	<2.5	Widely available	Good, extensive use reported, moderate reproducibility Recommended in guidelines	[S40]
	IMR	≥25			[S41]
	RRR	<3.5			[S42]
	MRR	3.0			[S43, 44]
Continuous thermodilution	CFR	<2.5	Research or academic centres	Increasingly investigated, high reproducibility Recommended in guidelines	[S45–48]
	Absolute hyperaemic resistance	> 480 Woods Units			
	MRR	<2.1			[S44, 49, 50]
**Non-wire based**					
Angiography-based	IMR_angio_	≥25 ≥40 severe	Research or academic centres	Increasingly investigated Quantitative criteria established Not in current guidelines	[S51, 52]
**B. Myocardial bridging assessment**					
Invasive coronary angiography	Nil	Milking effect	Widely available	Increasingly investigated Quantitative criteria established Not in current guidelines	[S53]
Intracoronary imaging	IVUS OCT	Half-moon sign Fusiform, signal poor border with systolic compression Cross-sectional area & phase of cardiac cycle >10% systolic compression	Selected centres		
Invasive coronary physiology	Doppler wire FFR dFFR iFR WIA	Fingertip sign ≤0.75 ≤0.76 ≤0.85	Selected centres		
**C. VSA assessment**					
Invasive	Acetylcholine (or ergot or hyperventilation)	Transient (sub)total coronary artery occlusion (>90% constriction) with: Angina Ischaemic ECG changes	Widely available	COVADIS criteria established Recommended in guidelines	[S54]

Non-invasive assessments						
Modality	Stressor	Metric	Threshold	Clinical availability	Utility in RA	Reference
**A. CMD assessment**						
**ECG**	Exercise	ST-segment depression	≥0.1-mV ST-segment depression 80ms from the J-point on ECG	Research or academic centre	Widely available but limited evidence.	[S55]
**CPET**	Exercise	MVO_2_	Peak MVO_2_ 17.3 vs 27.3 mL/kg/min in normal controls	Research or academic centre	Available but limited evidence.	[S56]
**Stress echocardiography**						
Wall motion assessment	Exercise Dobutamine Adenosine Dipyridamole	WMSI	Low sensitivity (44%) and specificity (56.1%)	Routine	Widely available but limited evidence	[S57]
LAD Doppler	Adenosine Dipyridamole	CFVR	CFVR < 2	Research or academic centre	Available and increasing evidence	[S58]
*MCE perfusion*	Adenosine Dipyridamole Regadenoson	Refill time	>2 s during vasodilator stress	Research or academic centre	Investigational	[S59]
		Stress MBF Microvascular flux rate (β) β reserve	236 intensity units/sec 1.6/sec 1.95			[S60]
**Nuclear**						
SPECT (^99m^Tc-sestabmibi, ^99m^Tc-tetrofosmin)	Adenosine Regadenoson	Qualitative & summed scores	-	Routine	Widely available but limited evidence	
Dynamic SPECT	Adenosine Regadenoson	MBF MFR	-	Research or academic centre	Limited availability and evidence	
PET (^15^O, ^82^Rb, ^13^NH)	Adenosine Dipyridamole Regadenoson	CFR	CFR < 2	Research or academic centre	Limited availability but recommended	[S61, 62]
**Perfusion CMR**						
Visually adjudicated	Adenosine Regadenoson	Visual perfusion defect	Circumferential subendocardial perfusion defect	Routine	Available but limited evidence	[S63]
Semi-quantitative	Adenosine, Regadenoson	MPRI	1.84	Research or academic centre	Available but limited evidence	[S64]
Quantitative	Adenosine, Regadenoson	MPR Stress MBF Endo:epi ratio	<2.4 <1.82	Research or academic centre	Limited availability but recommended	[S33, 65]
**B. Myocardial bridging assessment**						
Stress echocardiography	Exercise	Visual	Focal septal buckling with apical scarring	Research or academic centre	Available but limited evidence	[S66, 67]
CT	Nil	mm of overlying myocardium MMI (depth × length of muscle bridge)	≥2mm: ‘deep muscle bridge’ ≥5mm: ‘very deep muscle bridge’ MMI ≥31 predicted abnormal dFFR ≤0.76 with 74% sensitivity and 62% specificity	Research or academic centre	Available but limited evidence	[S53, 68]
		FFR-CT	≤0.75 (Gray zone 0.75–0.80)			
**C. VSA assessment**						
ECG	Ambulatory or at time of symptoms	ST segment changes	ST segment elevation ≥0.1mV ST segment depression≥0.1mV New negative U waves	Selected centres	Available and recommended	[S69, 70]

AChFR, acetylcholine flow reserve; ANOCA, angina and non-obstructed coronary arteries; CFR, coronary flow reserve; CFVR, coronary flow velocity reserve; COVADIS, Coronary Vasomotor Disorders International Study Group; CMR, cardiac MRI; CT, computed tomography; dFFR, diastolic FFR; ECG, electrocardiogram; endo:epi ratio, endocardial to epicardial ratio; FFR, fractional flow reserve; hMR, hyperaemic microvascular resistance; iFR, instantaneous free wave ratio; IMR, index of microcirculatory resistance; IMR_angio_, angiography-derived IMR; INOCA, ischaemia and non-obstructed coronary arteries; IVUS, intravascular ultrasound; LAD, left anterior descending; MBF, myocardial blood flow; MCE, myocardial contrast echocardiography; mm, millimetres; MMI, myocardial bridge muscle index; MPR, myocardial perfusion reserve; MPRI, myocardial perfusion reserve index; MRI, magnetic resonance imaging; MRR, microvascular resistance reserve; MVO_2_, maximum oxygen uptake; OCT, optical coherence tomography; PET, positron emission tomography; Pzf, pressure at zero flow; RA, refractory angina; RRR, resistive reserve ratio; VSA, vasospastic angina; WIA, wave intensity analysis; WMSI, wall motion score index. *Specific cut-off values for each modality are subject to ongoing investigation.*

### Wire-based epicardial assessments—measurements of coronary pressure

Wire-based, haemodynamic lesion assessment has a Class IA indication in guidelines.^36^ Various hyperaemic and non-hyperaemic indices are available (*Table 2*) with detailed procedural protocols reported elsewhere.^41,42^ In refractory angina, epicardial physiology assessment may be particularly helpful by revealing a diffuse atherosclerosis phenotype, which is unsuitable for conventional revascularization (*Table 2* and *Figure 2*).

### Assessment of myocardial bridging

Myocardial bridging is often identified incidentally on CT or invasive coronary angiography. Specific attribution of patients’ symptoms to the presence of bridging is challenging. Coronary flow insufficiency can be explained by reduced accelerating wave energy in early systole due to compression by myocardial bridging but may also be explained by CMD, endothelial dysfunction, and vasospasm, which frequently co-exist.^43^ Invasive and non-invasive diagnostic markers of bridging severity are shown in *Table 3*. The lack of reporting of the performance of these metrics in systematic studies may explain the lack of specific criteria for diagnosis and stratification of lesions requiring treatment in guidelines. Invasive wire-based haemodynamic and vasospasm testing should be considered in patients with refractory angina attributed to significant bridging.^44,45^ Testing should involve evaluation of bridging severity after dobutamine and exercise stress as well as exclusion of CMD and vasospasm.^43,46^

Clinical judgement needs to be exercised when selecting patients who require treatment of bridging, taking into account factors such as the presence and severity of ischaemia, extent of epicardial CAD, presence of cardiomyopathy, valvular heart disease or left ventricular hypertrophy.^46^

### Acetylcholine provocation testing for coronary artery spasm

Invasive assessment of epicardial or microvascular vasospasm using intracoronary acetylcholine is safe and is recommended in both European and US guidelines using the COVADIS criteria.^30,36,47^ The protocols have been reviewed previously.^28,48,49^ Vasospasm can co-exist with other ischaemic mechanisms such as CMD^50,51^ or after previous revascularization^52^ and should be considered as a cause of refractory angina.

### Wire-based assessments of microvascular function—measurement of coronary flow and pressure

Measurements of coronary flow use either Doppler or thermodilution techniques at rest and hyperaemia, enabling calculation of CFR.^42^ Of these, Doppler blood velocity and continuous thermodilution measurement offer the greatest reproducibility (*Table 3*). Additionally, measurements of microvascular resistance can be obtained enabling identification of functional and structural endotypes.^41^

### Angiography-derived assessments

Wire-based assessments of coronary physiology incur additional cost, expertise, are not ubiquitously available, and are underutilized.^53^ Derivation of metrics of both epicardial and microvascular physiology solely from coronary angiograms is attracting increasing interest (*Tables 2* and *3*).^54,55^ Clinical use of angiography-derived physiology should be evaluated further in patients with refractory angina, pending further clinical validation and demonstration of reproducibility.

## Non-invasive approach to coronary artery and ischaemia assessment

Non-invasive imaging modalities are established for adjudication of reversible regional ischaemia.^36,47^ These methods are particularly advantageous in patients with refractory angina by enabling simultaneous evaluation of the presence and transmurality of ischaemia in multiple coronary territories and interrogating myocardium subtended by coronary anatomy unsuitable for invasive assessment. This is most often reported by visual assessment.

Few studies have investigated the utility of individual diagnostic modalities to detect ischaemia in refractory angina. Myocardial blood flow (MBF) quantification may be especially beneficial in patients with refractory angina by enabling detection of balanced ischaemia or CMD by quantifying MBF responses to vasodilators (*Figure 2*).^56–58^ Potential metrics are summarized in *Tables 2* and *3*, though technique and metric-specific cut-offs for each are being established.^59–61^ Non-invasive imaging methods to evaluate coronary vasospasm and myocardial bridging have been reported but are not currently recommended by guidelines.^46,62^

Given the potential importance of quantitative MBF assessment to evaluate refractory angina, the discussion focuses on currently available quantitative methods. Research is ongoing to define which parameters provide the greatest clinical utility for diagnosing obstructive epicardial CAD, CMD, or their combination in patients both chronic coronary syndromes and refractory angina.

### Positron emission tomography

Positron emission tomography (PET) is the reference standard for non-invasive quantitative assessment of myocardial perfusion.^63^ The added utility of quantitative PET perfusion assessment has been demonstrated in advanced epicardial CAD^61,64,65^ as well as CMD.^66^ PET imaging quantifies regional and global MBF at rest and after vasodilator stress (mL/min/g), the ratio of which provides CFR (Stress/Rest MBF). Coronary flow reserve by PET also provides prognostic information for risk stratification of future adverse cardiovascular events.^67–71^

Quantitative metrics integrating stress MBF and CFR, such as coronary flow capacity, are gathering interest.^72–74^ Preliminary studies have compared PET with invasive coronary blood flow assessment.^75,76^ Further work using artificial intelligence will enable more granular understanding of regional myocardial blood flow variations and classification of CAD and CMD endotypes.^74^

### Stress perfusion cardiac MRI

With increasing availability combined with high sensitivity and specificity to detect myocardial ischaemia, stress perfusion cardiac MRI (CMR) is an effective modality to assess patients with obstructive epicardial CAD.^77^ In refractory angina, CMR may be particularly advantageous as it can simultaneously characterize myocardial contractile function and fibrosis. Quantitative myocardial perfusion assessment to provide absolute MBF values is feasible and increasingly available.^78,79^ These software solutions generate fully automated segmentation and quantitative parametric maps and have been used in patients with epicardial CAD and CMD.^59,80^ Cardiac MRI may be suited to evaluate ischaemia in refractory angina where both epicardial and microvascular ischaemic mechanisms co-exist. Ongoing research aims to define diagnostic thresholds for different ischaemic mechanisms, which will establish the clinical utility of quantitative perfusion CMR in refractory angina.

### Doppler coronary flow velocity reserve

Transthoracic Doppler echocardiography of the left anterior descending artery can measure coronary blood flow velocity at rest and during pharmacological hyperaemia enabling calculation of a coronary flow velocity reserve.^81–83^ This method has a Class IIb, level of evidence B recommendation to assess CMD.^36^ However, its use in clinical practice is not common due to the need for specific training; operator-dependency; assessment restricted to the left anterior descending (excluding other native or revascularized coronary territories); and limited acoustic windows. Stress myocardial contrast echocardiography may enable quantitative regional perfusion assessment and offer additional benefits.^84,85^

## Pharmacological treatment to improve symptoms—a stratified approach

The evidence base for effective pharmacologic treatments in patients with refractory angina is limited (*Table 4* and Supplementary data online, *Table S3*). In these patients, multiple mechanisms of ischaemia often co-exist resulting in symptoms, which remain refractory to initial empiric approaches to pharmacologic treatment which usually focus on a single perceived mechanism of ischaemia. Implementation of personalized treatment stratified according to the underlying ischaemic mechanisms may be a more effective strategy for addressing refractory symptoms.

**Table 4:** Pharmacological options for anti-ischaemic therapy

Drug	Mechanism of action	Evidence of efficacy			
		Stable angina/obstructive epicardial CAD	Coronary microvascular dysfunction	Vasospasm	Refractory angina
**Beta-blockers**	Reduce heart rate and myocardial oxygen demand Increase diastolic filling time Reduce afterload	Class I recommendation [S71–73]	Recommended in ESC and AHA/ACC guidelines [S71, 72, 74]	Concomitant use of β-blockers for vasospastic angina can be considered without significant epicardial coronary stenosis (Class IIb) [S75] After DES implantation, no evidence of increased frequency of ACh-induced vasospasm with beta-blocker therapy [S76, 77].	No study
**Vasodilating beta-blockers**	As for beta blockers Vasodilatation via alpha blockade & NO generation	Carvedilol: Improvement in exercise tolerance, time to onset of angina, and 1 mm ST-segment depression [S78] Nebivolol: increased ischaemic and anginal thresholds [S79]	Suggested in AHA/ACC guidelines [S72] *Results of NIRVANA Trial awaited*	As above	No study
**Calcium channel blockers**	Reduce heart rate and myocardial oxygen demand Vasodilation via action on vascular smooth muscle Reduce afterload	Class I recommendation [S71, 72]	Amlodipine: Improvement in exercise time (ChaMP-CMD) [S80]	Amlodipine reduces rate of angina episodes [S81] Diltiazem—EDIT-CMD trial improves epicardial vasospasm on coronary function testing [S82]	No study
**Long-acting nitrates**	Vasodilation via action on smooth muscle Reduce preload	Class IIa, Level of evidence B [S71]	Limited evidence Limited benefit due to small vasodilatory effect on small resistance vessels [S103].	Reduction in angina frequency [S104]. Did not improve long-term prognosis in patients when combined with CCBs [S105].	No study
**Nicorandil**	Cytoprotective effects Vasodilation through NO donation	IONA Trial [S83] ESC 2024 Class IIb, level of evidence B [S71]	Limited evidence with small number of studies in CMD [S84–86]	Limited evidence Reduction in ergometrine-induced coronary spasm [S87]	No RCT Limited evidence with small studies in RA [S88]
**Metabolic modifiers**					
**Ranolazine**	Inhibition of late inward sodium current Improves ionic homeostasis and myocardial energetics Reduce myocardial oxygen demand	ESC 2024 Class IIa, level of evidence B recommendation [S71] CARISA Trial [S89] RIVER-PCI [S90]	Improvement in exercise time [S80] Improvements in symptoms, quality of life, exercise performance and CFR [S91, 92] Varying reports of effect on SAQ angina [S93, 94] No improvement in symptoms or microvascular function (MARINA Trial) [S95]	No study	No RCT Symptomatic improvement in observational cohort studies, no RCT (Ranolazine Refractory Angina Registry) & Ling *et al*. [S96, 97]
**Trimetazidine**	Partial inhibition of β−oxidation & increases glucose oxidation Increases cellular tolerance to ischaemia	ESC 2024 Class IIb, level of evidence B [S71] AT-PCI trial [S98] PATMOS trial [S99] Meta-analysis of 13 studies showing clinical efficacy [S100]	Limited evidence Improved total exercise time, time to 1 mm ST-segment depression and maximum ST-segment depression [S101] Another study failed to show benefit [S102]	No additional benefit on clinical outcomes when added to diltiazem and nitrates [S103]	No RCT Meta-analysis showed improvements in walking time and angina severity in patients not suitable for revascularisation [S104]
**Ivabradine**	Reduce heart rate through inhibition of *I_f_*	ESC 2024 Class IIa, level of evidence B, for LVEF < 40% and SR > 70bpm [S71] BEAUTIFUL & SIGNIFY Trials [S105, 106]	Improvement in SAQ [S94, 107] No improvement in time to 1 mm ST-segment depression or effect on microvascular function [S94]	No study	No study
**L-arginine**	Substrate for NO synthase Improve endothelium-dependent vasodilation	Limited evidence [S108]	Limited evidence Suggested improvements in endothelial function	Limited evidence Long-term supplementation improved small-vessel endothelial function with improvement and symptoms [S109]	No study
**Rho-kinase inhibitors**	Coronary vasodilation	Limited evidence Improvements on treadmill exercise test observed [S110]	Limited evidence Improvement in microvascular resistance in patients also with VSA [S111]	Improvement in ischaemia in microvascular spasm [S112, 113]	No study
**ACEi/ARB**	Inhibition of the effects of angiotensin II Improves endothelial function	No study on its anti-anginal effect	CorMiCA [S114] WISE Substudy [S115] AHA/ACC 2023 Table 17 [S72]	Limited evidence Suggested improvements in angina when combined with CCB [S116]	No study

ACEi, angiotensin-converting enzyme inhibitors; ACh, acetylcholine; AHA, American Heart Association; ACC, American College of Cardiology; bpm, beats per minute; ARB, angiotensin receptor blocker; CAD, coronary artery disease; CCB, calcium channel blocker; CFR, coronary flow reserve; cGMP, guanosine 3’, 5’-cyclic monophosphate; CMD, coronary microvascular dysfunction; DES, drug-eluting stent; ESC, European Society of Cardiology; ET:1, endothelin-1; I*_f_*, funny current; LVEF, left ventricular ejection fraction; NO, nitric oxide; RA, refractory angina; RCT, randomized controlled trial; SAQ, Seattle Angina Questionnaire; SR, sinus rhythm; VSA, vasospastic angina.

### Epicardial CAD

Selection of drug therapy according to the ‘diamond’ approach stratified by heart rate, blood pressure, left ventricular function and co-morbidities is recommended (*Table 4*).^36,86^ Few placebo-controlled randomized trials (RCT) evaluate anti-anginal drugs specifically in refractory angina,^87,88^ requiring extrapolation from conventional treatment algorithms for stable angina.

### Coronary microvascular dysfunction

The optimal pharmacological treatment of CMD remains undefined. The stratified medical therapy approach has been established by the CorMiCA trial,^50,89^ which is associated with improved symptoms and quality of life at 1 year. A double-blinded, crossover RCT has suggested that only patients with an invasive CFR < 2.5 improved their symptoms and exercise performance with ranolazine or amlodipine.^90^ However, a significant burden of refractory angina often remains.^89,90^ In a small study, trimetazidine prolonged total exercise time and time to 1 mm ST depression compared with placebo.^91^ Contemporary studies of non-dihydropyridine calcium-channel blockers and selective endothelin A receptor antagonists, have not demonstrated clinical benefit.^52,92^ Other investigational drugs are summarized in Supplementary data online, *Table S3*.

### Myocardial bridging

Currently, there are no RCTs for the pharmacological treatment of myocardial bridging. Beta-blockers are considered first-line therapy.^46^ An endothelium-dependent vasodilating beta-blocker such as nebivolol may offer additional benefit.^43^ Calcium-channel blockers can be considered as alternatives, particularly when associated with coronary vasospasm. Many patients may have co-existing endothelial dysfunction and CMD which should be treated.^12^

### Vasospasm

Guideline recommended and investigational treatments for coronary vasospasm are summarized in *Table 4* and Supplementary data online, *Table S3*, respectively. Coronary vasospasm can arise from endothelial dysfunction or smooth muscle hyper-reactivity.^93^ Calcium-channel blockers are considered first-line for epicardial and microvascular spasm.^48,94,95^ For persistent symptoms, nicorandil or long-acting nitrates should be additionally considered. The latter can be associated with tolerance and rebound vasoconstriction after medication discontinuation and may worsen endothelial dysfunction.^96^ However, 20% of patients may have refractory symptoms despite these first-line therapies demonstrating the need to consider additional treatment options. Rho-kinase inhibitors (*Table 4*) and denopamine (see Supplementary data online, *Table S3*) may help in patients with refractory symptoms.^48,94,97,98^ Medications which improve endothelial function can be considered.^94^ Recent studies have shown no benefit of endothelin receptor antagonists to improve angina in patients with coronary vasospasm (see Supplementary data online, *Table S3*).

## Non-pharmacological treatment—a stratified approach

**Figure 3:**
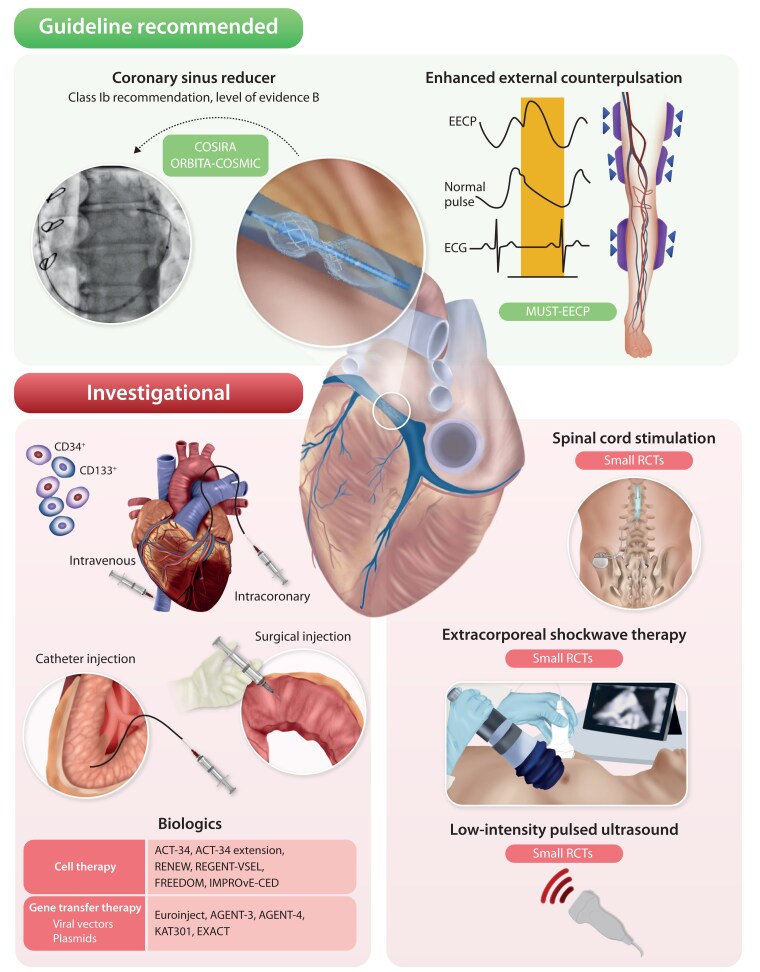
Non-pharmacological anti-ischaemic therapies for patients with refractory angina

Various non-pharmacological options to relieve ischaemia and improve angina in refractory angina have been evaluated (*Figure 3*, Supplementary data online, *Table S4*).

### Coronary sinus reducer

In patients with refractory angina and reversible ischaemia, the coronary sinus reducer (CSR) may be considered to improve symptoms and quality of life. Conventionally, this has been recommended for patients with left coronary territory ischaemia. In the current guidelines, CSR is the only non-pharmacologic option recommended for refractory angina (Class IIb, Level of evidence B)^36^ based on the results of two double-blinded, sham-controlled RCTs, and a significant body of open label and registry data.^99–101^ The angina improvement observed in COSIRA has been confirmed in a second double-blinded sham-controlled RCT (ORBITA-COSMIC) that assessed angina using a patient-reported symptom app.^102^ Sustained symptom improvement out to two years follow-up has been shown in the REDUCER-1 registry.^103^ Coronary sinus reducer implantation is associated with a favourable peri-procedural and long-term safety profile. The underlying mechanism of action mediating these symptomatic improvements remains under investigation. Recent evidence suggests that CSR may redistribute blood flow to under-perfused myocardium, particularly in the subendocardium, through an effect on the microcirculation.^102,104,105^ Ongoing double-blinded, sham-controlled RCTs will provide further insights into the efficacy and mechanistic basis of angina improvement resulting from CSR in patients with refractory angina secondary to epicardial CAD (NCT05102019) and CMD (NCT05492110).

### Enhanced extracorporeal counterpulsation

Enhanced extracorporeal counterpulsation (EECP) is a non-invasive therapy with a Class IIb Level of evidence B recommendation in US guidelines.^106^ The procedure has been described elsewhere with preliminary evidence suggesting that EECP improves endothelial function and collateral flow.^107,108^ There are no RCTs of EECP in refractory angina. Its use is extrapolated from a RCT in patients with stable angina, which showed improvement in time to ST-segment depression and angina frequency.^109^ While these initial results are supported by a meta-analysis,^110^ a subsequent evaluation did not demonstrate clinical or cost-effectiveness.^111^ The potential benefits of EECP in patients with angina and unobstructed coronary arteries have been reported.^112^ Additional research is needed to define the role of EECP in refractory angina.

### Biologicals

For more than 20 years, various regenerative therapies, mainly administered by an intracoronary or intramyocardial route, have been evaluated in refractory angina.^3,113,114^ Of these, selected bone marrow–derived CD34 + progenitor cells have been studied most and were evaluated in a meta-analysis showing reduced angina frequency and improved exercise time compared to placebo.^115^ Recent studies have also shown improvements in endothelial function and CFR after administration of autologous CD34 + cells in patients with refractory angina secondary to CMD.^116,117^ A recent phase 2 trial of trans-epicardial delivery of an adenoviral-5 vector expressing vascular endothelial growth factor isoforms has demonstrated safety and signals of improved exercise duration and reduced ischaemic burden up to 12 months.^118^ Biologic treatment strategies aimed at neovascularization of ischaemic myocardium in refractory angina remain investigational.

### Extracorporeal shockwave therapy

Extra-corporeal shockwave therapy (ECSWT) is a non-invasive therapy which delivers low energy shockwaves targeted to ischaemic myocardium. Experimental studies indicate that ECSWT promotes collateral development and capillary density resulting in improved myocardial perfusion.^119^ Trials investigating ECSWT in refractory angina have shown improvements in myocardial perfusion and quality of life.^120,121^ Data also suggest a sustained symptomatic improvement with ECSWT over ∼3 years. However, ECSWT is not recommended in guidelines and further research is needed to define mechanism and confirm clinical efficacy.

### Low-intensity pulsed ultrasound

Low-intensity pulsed ultrasound therapy is a potential non-invasive investigational therapy to help patients with refractory angina. In a porcine model, low-intensity pulsed ultrasound promoted angiogenesis and upregulated vascular endothelial growth factor, endothelial nitric oxide synthase and basic fibroblast growth factor within ischaemic myocardium.^122^ A recent double-blinded, placebo-controlled RCT did not demonstrate improvements in nitroglycerin use compared to placebo, although a trend towards anti-ischaemic effects was observed.^123^ Further studies are needed to confirm its utility to treat refractory angina.

### Non-pharmacological options for myocardial bridging

In patients with myocardial bridging in whom angina persists despite pharmacological treatment, additional options such as PCI within the bridged segment, surgical unroofing, or CABG have been suggested. Few systematic evaluations report the clinical benefit and relative risk-benefits of these approaches, which would be needed to stratify patients towards a specific treatment pathway.^124–126^ Only symptomatic patients in whom the pathophysiological significance of the bridging has been confirmed, and co-existent CMD and vasospasm have been excluded or treated, should be considered for these options.

## Advanced pain management

For patients who continue to experience life-limiting angina despite stratified anti-ischaemic therapy, more sophisticated pharmacological, non-pharmacological, and behavioural pain management should be considered (see Supplementary data online, *Figure S1*). Neuro-humoral interactions between the heart and the central nervous system, triggered by myocardial ischaemia, are the foundation of angina perception.^127–129^ During ischaemia, myocytes release algogenic agents such as adenosine and substance P that stimulate afferent sympathetic cardiac neurons. These afferent nociceptive signals are transmitted centrally via the stellate ganglion to the thoracic spinothalamic tracts.^129,130^ Signalling via this neuro-cardiac axis can be modulated at multiple levels (see Supplementary data online, *Figure S1*).

Evidence for the use of analgesic drugs in refractory angina is limited (see Supplementary data online, *Table S5*). Whilst these approaches have not been specifically tested in refractory angina, it may be plausible to consider their use to reduce anginal symptoms, supervised by a specialist pain clinic, based on their reported efficacy in chronic pain syndromes.^131^

Non-pharmacological treatments are summarized in Supplementary data online, *Table S5*. Limited data exist for neuromodulation by transcutaneous (TENS) or subcutaneous electrical nerve stimulation (SENS), which are predominantly used to screen patient eligibility for spinal cord stimulation.^1^ Spinal cord stimulation has the most evidence for efficacy in refractory angina, but studies were small and the results inconsistent (see Supplementary data online, *Table S5*). The ongoing placebo-controlled, double-blinded, crossover SCRAP RCT will clarify the role of spinal cord stimulation in refractory angina.^132^ Sympathectomy has been used to manage chronic pain syndromes (see Supplementary data online, *Table S5*). In refractory angina, temporary stellate ganglion block did not significantly improve angina compared to placebo. No data are available for the efficacy of permanent sympathectomy in refractory angina.

Finally, cognitive behavioural therapy can modify patients’ chronic maladaptive responses and health beliefs resulting from recurrent angina. This can be delivered either face-to-face or via online platforms and may improve anginal symptoms, psychological morbidity, and quality of life (see Supplementary data online, *Table S5*). These programs may be best delivered together with CR and can be considered for patients with refractory angina.

## Risk factor management and cardiac rehabilitation

Patients across the spectrum of refractory angina have multiple modifiable adverse cardiovascular risk factors. For those with established epicardial CAD, guidelines define specific treatment targets to modify risk factors, which are associated with improved major adverse cardiovascular events.^133^ Patients with CMD and vasospasm frequently have early atheromatous change in their coronary arteries. Furthermore, CMD and vasospasm can co-exist with established CAD. Therefore, while not formally supported by outcomes data, it would seem biologically plausible to treat cardiovascular risk factors in refractory angina patients to the targets established in guidelines for epicardial CAD. Treatment with ACEi/ARBs may reduce major adverse cardiovascular events in patients with epicardial vasospasm.^134^ The prognostic benefit of intensive cardiovascular risk factor modification and anti-platelet therapy in ANOCA/INOCA remains under investigation.^135^ Implementation of systematic risk factor modification to attain guideline-directed targets in patients with refractory angina remains suboptimal.^136,137^ This may be improved by enrolment into formal CR programs that include optimization of cardiovascular risk factor control as a fundamental component.

Patients with refractory angina should also be considered for lifestyle interventions, in particular, smoking cessation which remains not only a leading cause of atherosclerosis worldwide but is also associated with vasospasm and CMD.^138–140^ A program of weight loss, low energy diet, and risk factor modification may improve angina control and microvascular function.^141^

A major barrier to CR is concern regarding its safety in patients with refractory angina. In those with obstructive CAD, exercise rehabilitation can be successfully delivered and improves physical ability.^142,143^ A meta-analysis has confirmed that CR reduces mortality, hospitalization, and improves quality of life in patients with CAD.^144^ Initial work suggests improvements in exercise capacity, quality of life, symptom burden, and ischaemia in ANOCA/INOCA.^145^ Recent consensus documents suggest beneficial effects of CR for patients with microvascular angina.^146,147^ Physical performance improves after CR in vasospasm.^148^ With increasing evidence of the wide-ranging benefits of CR across the spectrum of patients comprising the refractory angina population, future guidelines will place a greater emphasis on its role.

## A specialist angina heart team for the management of patients with refractory angina

**Figure 4:**
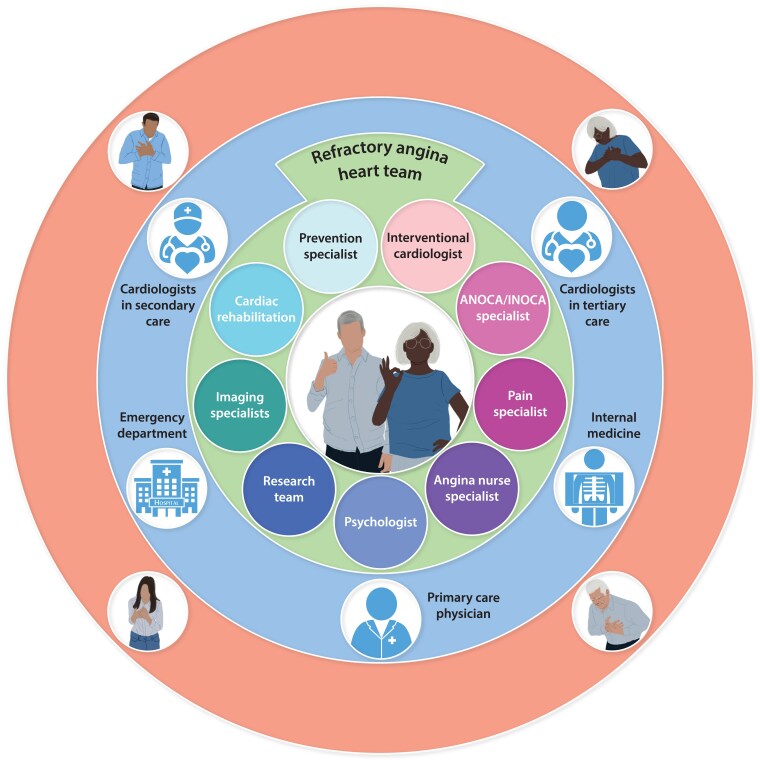
The multi-disciplinary Refractory Angina Heart Team. ANOCA: angina and non-obstructive coronary arteries, INOCA: ischaemia and non-obstructive coronary arteries

The essential elements needed to manage the complex and diverse clinical needs of refractory angina patients have been described. Effective delivery of each component should be tailored to individual patients and may be best delivered through an integrated specialist service. Building on previous suggestions^3,149,150^ a comprehensive service model designed to deliver patient-centred refractory angina management is proposed. Recognizing that multiple ischaemic mechanisms may co-exist (*Figure 1*), this service should offer the full range of diagnostic and stratified treatment pathways (*Figures 2 and 3*, *Tables 2–4)* described for both epicardial CAD and ANOCA/INOCA. The required facilities and personnel may not be available in all centres and propose that a hub-and-spoke model of referrals into a comprehensive multi-disciplinary service may provide a solution (*Figure 4*).

A care model with an angina nurse specialist at its centre, who is integrated within the wider multi-disciplinary team, may be effective (*Figure 4*). This provides patients with an accessible single point of contact with whom they can easily communicate their clinical needs and enables co-ordinated delivery of the specific components of their multi-disciplinary care. Furthermore, angina nurse specialists will be well placed to communicate care plans with referring physicians.

## Conclusion

With a growing population of patients across the full spectrum of chronic coronary syndromes, the proportion of patients with refractory symptoms will expand. In addition to the morbidity and significantly impaired quality of life these patients encounter, this growth in the refractory angina population will place an increasing burden on healthcare resources and associated care costs. Evidence-based therapies which effectively treat the spectrum of ischaemic mechanisms occurring in patients with refractory angina remain inadequate and highlight a major priority for future research. Effective deployment of currently available treatments requires confirmation of underlying myocardial ischaemic mechanisms, for example, routine evaluation for CMD and vasospastic angina in patients with persistent symptoms after revascularization, which enables the implementation of appropriate stratified therapy. These may be best offered within the context of a multi-disciplinary team that can manage the healthcare complexities experienced by refractory angina patients.

The assessment of angina pectoris and its management are complex. This article proposes an approach to navigate this complexity using structured and comprehensive investigation to inform selection of appropriate stratified treatments in order to reduce the population of patients whose symptoms remain refractory to treatment. Further evaluation of this patient-centred multi-disciplinary management strategy to achieve improved clinical outcomes for patients with refractory angina is warranted.

## Supplementary Material

ehaf284_Supplementary_Data

## Declarations

### Data Availability

No data were generated or analysed for or in support of this paper.
