# Supplementary material for: Refractory angina: mechanisms and stratified treatment in obstructive and non-obstructive chronic myocardial ischaemic syndromes
Source: Eur Heart J. 2025 Jul 1;46(38):3738–57. doi: 10.1093/eurheartj/ehaf284 (PMC12500330; doi:10.1093/eurheartj/ehaf284)
Supplement: ehaf284_Supplementary_Data [file ehaf284_supplementary_data.docx]

**Table 2. Invasive and non-invasive diagnostic approaches for obstructive epicardial coronary artery disease.**

| **Invasive assessments** | | | | | |
| --- | --- | --- | --- | --- | --- |
| **Modality** | **Metric** | **Cut-off** | **Clinical availability** | **Reproducibility** | **Reference** |
| **Wire-based** | | | | | |
| Hyperaemic | FFR | ≤0.80 | Widely available | +++  Recommended in guidelines | [S1] |
|  | CFR | <2.0 | Widely available |  | [S2, 3] |
|  | hSR | ≥0.80 Hg/cm s | Currently not available | +++ | [S4] |
|  | PPG | 0.73 | Research | +++ | [S5] |
| Non-hyperaemic | iFR  RFR  DFR  Whole-cycle Pd/Pa | ≤0.89  ≤0.91 | Widely available | +++  Recommended in guidelines | [S6, 7] |
|  |  |  |  | +++ |  |
| **Non-wire-based** | | | | | |
| Angiography-based | QFR | <0.80 | Selected centres | +++  Recommended in guidelines | [S8-14] |
|  | vFFR  caFFR  FFR_angio_ |  |  | ++ |  |

| **Non-invasive assessments** | | | | | | |
| --- | --- | --- | --- | --- | --- | --- |
| **Modality** | **Stressor** | **Metric** | **Threshold** | **Clinical availability** | **Reproducibility** | **Reference** |
| **ECG** | Exercise | ST-segment depression | ≥0.1mV horizontal or down-sloping ST-segment depression 80msec from J point | Routine | +/++ | [S15] |
| **Cardiopulmonary exercise testing** | Exercise | Peak VO_2_ | ECG criteria as per exercise ECG +  gas exchange analysis | Routine | ++ | [S16-21] |
|  |  | O_2_ pulse flattening |  |  |  |  |
|  |  | ΔVO2/Δwork rate slope |  |  |  |  |
| **Computed tomography** | N/A | Stenosis severity | 70% | Routine | ++ | [S22] |
|  |  | FFR-CT | ≤0.80 | Selected centres | +++ |  |
|  |  | Perfusion | MBF index <78ml/100ml/min | Research or academic centre | Investigational | [S23] |
| **Stress Echocardiography** | | | | | | |
| Wall motion assessment | Exercise  Dobutamine  Adenosine Dipyridamole | WMSI | New or worsening stress-induced segmental regional wall motion or thickening | Routine | ++ | [S24] |
| LAD Doppler | Adenosine Dipyridamole | CFVR | CFVR <2 | Selected centres | +/++ | [S25] |
| MCE perfusion | Adenosine Dipyridamole | Refill time | >2 secs during vasodilator stress | Research or academic centre | Investigational | [S26] |
| **Nuclear** | | | | | | |
| SPECT  (^99m^Tc-sestabmibi, ^99m^Tc-tetrofosmin) | Exercise  Adenosine  Dipyridamole  Regadenoson | Qualitative & summed scores | Qualitative or semiquantitative grading of reversible perfusion defect on rest/stress imaging | Routine | ++ | [S27] |
| Quantitative SPECT | Exercise  Adenosine  Dipyridamole  Regadenoson | MBF  MFR | Stress MBF 1.86ml/min/g  MFR 1.61-1.95 | Research or academic centre | +++ | [S28, 29] |
| PET  (^15^O, ^82^Rb, ^13^NH, ^18^F-flurpiridaz) | Adenosine  Dipyridamole  Regadenoson | CFR  *CFC* | Qualitative or semiquantitative grading of reversible perfusion defect on rest/stress imaging  CFR <2  *Graded severity thresholds* | Research or academic centre | +++ | [S30] |
| **Perfusion CMR** | | | | | | |
| Visual adjudicated | Adenosine Regadenoson | Visual perfusion defect | Segmental transmurality and persistence of stress perfusion defect compared with rest perfusion or late gadolinium enhancement | Routine | ++ | [S31] |
| Semi-quantitative | Adenosine, Regadenoson | MPRI | <1.1 | Research or academic centre | ++ | [S32] |
| Quantitative | Adenosine, Regadenoson | MPR  Stress MBF  Endo:Epi ratio | Stress MBF <1.94 | Research or academic centre | +++ | [S33, 34] |
| CAD: coronary artery disease; caFFR: computation pressure-flow dynamics derived FFR; CFC: coronary flow capacity; CFR: coronary flow reserve; CFVR: coronary flow velocity reserve; CMR: cardiac MRI; DFR: diastolic hyperaemia-free ratio; ECG: electrocardiogram; endo:epi ratio: endocardial to epicardial ratio; FFR: fractional flow reserve; FFR_angio_: coronary angiography-derived FFR; FFR-CT; fractional flow reserve – computed tomography; hSR: hyperaemic stenosis resistance; iFR: instantaneous wave-free ratio; LAD: left anterior descending; MBF: myocardial blood flow; MCE: myocardial contrast echocardiography; MFR: myocardial flow reserve; MPR: myocardial perfusion reserve; MPRI: myocardial perfusion reserve index; MRI: magnetic resonance imaging; Pa: aortic pressure; Pd: distal coronary pressure; PET: positron emission tomography; PPG: pullback pressure gradient; QFR: quantitative flow ratio; RFR: resting full-cycle ratio; SPECT: single-photon emission computed tomography; VCO_2_: volume of carbon dioxide production; VE: minute ventilation; vFFR: vessel FFR; VO_2_: volume of oxygen uptake; WMSI: wall motion score index. Reproducibility: low (+), acceptable (++), good (+++).  *Specific cut-off values for each modality are subject to ongoing investigation.* | | | | | | |

**Table 3.** **Invasive and non-invasive diagnostic approaches for evaluation of ischaemia not attributable to obstructive epicardial coronary artery disease (fully referenced in Supplementary Appendix).**

| **Invasive assessments** | | | | | |
| --- | --- | --- | --- | --- | --- |
| **Modality** | **Metric** | **Cut-off** | **Clinical availability** | **Utility** | **Reference** |
| 1. **CMD assessment** | | | | | |
| **Wire-based** | | | | | |
| Intracoronary Doppler | CFR | <2.5 | Currently unavailable | - High and extensively validated - Recommended in guidelines | [S35-37] |
|  | hMR | >2.5 |  |  | [S35, 36] |
|  | Pzf | ≥42mmHg |  |  | [S38] |
|  | AChFR | ≤1.5 |  |  | [S39] |
| Bolus thermodilution | CFR | <2.5 | Widely available | - Good, extensive use reported, moderate reproducibility - Recommended in guidelines | [S40] |
|  | IMR | ≥25 |  |  | [S41] |
|  | RRR | <3.5 |  |  | [S42] |
|  | MRR | 3.0 |  |  | [S43, 44] |
| Continuous thermodilution | CFR | <2.5 | Research or academic centres | - Increasingly investigated, high reproducibility - Recommended in guidelines | [S45-48] |
|  | Absolute hyperaemic resistance | > 480 Woods Units |  |  |  |
|  | MRR | <2.1 |  |  | [S44, 49, 50] |
| **Non-wire based** | | | | | |
| Angiography-based | IMR_angio_ | ≥25  ≥40 severe | Research or academic centres | - Increasingly investigated - Quantitative criteria established - Not in current guidelines | [S51, 52] |

| 1. **Myocardial bridging assessment** | | | | | |
| --- | --- | --- | --- | --- | --- |
| Invasive coronary angiography | Nil | Milking effect | Widely available | - Increasingly investigated - Quantitative criteria established - Not in current guidelines | [S53] |
| Intracoronary imaging | IVUS  OCT | Half-moon sign  Fusiform, signal poor border with systolic compression  Cross-sectional area & phase of cardiac cycle  >10% systolic compression | Selected centres |  |  |
| Invasive coronary physiology | Doppler wire  FFR  dFFR  iFR  WIA | Fingertip sign  ≤0.75  ≤0.76  ≤0.85 | Selected centres |  |  |

| 1. **VSA assessment** | | | | | |
| --- | --- | --- | --- | --- | --- |
| Invasive | Acetylcholine  (or ergot or hyperventilation) | Transient (sub)total coronary artery occlusion (>90% constriction) with:   - Angina - Ischaemic ECG changes | Widely available | - COVADIS criteria established - Recommended in guidelines | [S54] |

| **Non-invasive assessments** | | | | | | |
| --- | --- | --- | --- | --- | --- | --- |
| **Modality** | **Stressor** | **Metric** | **Threshold** | **Clinical availability** | **Utility in RA** | **Reference** |
| 1. **CMD assessment** | | | | | | |
| **ECG** | Exercise | ST-segment depression | ≥0.1-mV ST-segment depression 80ms from the J-point on ECG | Research or academic centre | Widely available but limited evidence. | [S55] |
| **CPET** | Exercise | MVO_2_ | Peak MVO_2_ 17.3 vs. 27.3 ml/kg/min in normal controls | Research or academic centre | Available but limited evidence. | [S56] |
| **Stress Echocardiography** | | | | | | |
| Wall motion assessment | Exercise  Dobutamine  Adenosine Dipyridamole | WMSI | Low sensitivity (44%) and specificity (56.1%) | Routine | Widely available but limited evidence | [S57] |
| LAD Doppler | Adenosine Dipyridamole | CFVR | CFVR < 2 | Research or academic centre | Available and increasing evidence | [S58] |
| *MCE perfusion* | Adenosine Dipyridamole  Regadenoson | Refill time | > 2 secs during vasodilator stress | Research or academic centre | Investigational | [S59] |
|  |  | Stress MBF  Microvascular flux rate (β)  β reserve | 236 intensity units/sec  1.6/sec  1.95 |  |  | [S60] |
| **Nuclear** | | | | | | |
| SPECT  (^99m^Tc-sestabmibi, ^99m^Tc-tetrofosmin) | Adenosine  Regadenoson | Qualitative & summed scores | - | Routine | Widely available but limited evidence |  |
| Dynamic SPECT | Adenosine  Regadenoson | MBF  MFR | - | Research or academic centre | Limited availability and evidence |  |
| PET  (^15^O, ^82^Rb, ^13^NH) | Adenosine  Dipyridamole  Regadenoson | CFR | CFR < 2 | Research or academic centre | Limited availability but recommended | [S61, 62] |
| **Perfusion CMR** | | | | | | |
| Visually adjudicated | Adenosine Regadenoson | Visual perfusion defect | Circumferential subendocardial perfusion defect | Routine | Available but limited evidence | [S63] |
| Semi-quantitative | Adenosine, Regadenoson | MPRI | 1.84 | Research or academic centre | Available but limited evidence | [S64] |
| Quantitative | Adenosine, Regadenoson | MPR  Stress MBF  Endo:epi ratio | <2.4  <1.82 | Research or academic centre | Limited availability but recommended | [S33, 65] |
| 1. **Myocardial bridging assessment** | | | | | | |
| Stress echocardiography | Exercise | Visual | Focal septal buckling with apical scarring | Research or academic centre | Available but limited evidence | [S66, 67] |
| CT | Nil | mm of overlying myocardium  MMI  (depth x length of muscle bridge) | ≥2mm: “deep muscle bridge”  ≥5mm: “very deep muscle bridge”  MMI ≥31 predicted abnormal dFFR ≤0.76 with 74% sensitivity and 62% specificity | Research or academic centre | Available but limited evidence | [S53, 68] |
|  |  | FFR-CT | ≤0.75  (Gray zone 0.75-0.80) |  |  |  |
| 1. **VSA assessment** | | | | | | |
| ECG | Ambulatory or at time of symptoms | ST segment changes | ST segment elevation ≥0.1mV  ST segment depression≥0.1mV  New negative U waves | Selected centres | Available and recommended | [S69, 70] |
| AChFR: acetylcholine flow reserve; ANOCA: angina and non-obstructed coronary arteries; CFR: coronary flow reserve; CFVR: coronary flow velocity reserve; COVADIS: Coronary Vasomotor Disorders International Study Group; CMR: cardiac MRI; CT: computed tomography; dFFR: diastolic FFR; ECG: electrocardiogram; endo:epi ratio: endocardial to epicardial ratio; FFR: fractional flow reserve; hMR: hyperaemic microvascular resistance; iFR: instantaneous free wave ratio; IMR: index of microcirculatory resistance; IMR_angio_: angiography-derived IMR; INOCA: ischaemia and non-obstructed coronary arteries; IVUS: intravascular ultrasound; LAD: left anterior descending; MBF: myocardial blood flow; MCE: myocardial contrast echocardiography; mm: millimetres; MMI: myocardial bridge muscle index; MPR: myocardial perfusion reserve; MPRI: myocardial perfusion reserve index; MRI: magnetic resonance imaging; MRR: microvascular resistance reserve; MVO_2_: maximum oxygen uptake; OCT: optical coherence tomography; PET: positron emission tomography; Pzf: pressure at zero flow; RA: refractory angina; RRR: resistive reserve ratio; VSA: vasospastic angina; WIA: wave intensity analysis; WMSI: wall motion score index. *Specific cut-off values for each modality are subject to ongoing investigation.* | | | | | | |

**Table 4. Pharmacological options for anti-ischaemic therapy.**

| **Drug** | **Mechanism of action** | **Evidence of efficacy** | | | |
| --- | --- | --- | --- | --- | --- |
|  |  | **Stable angina/obstructive epicardial CAD** | **Coronary microvascular dysfunction** | **Vasospasm** | **Refractory angina** |
| **Beta-blockers** | Reduce heart rate and myocardial oxygen demand  Increase diastolic filling time  Reduce afterload | Class I recommendation  [S71-73] | Recommended in ESC and AHA/ACC guidelines  [S71, 72, 74] | Concomitant use of β-blockers for vasospastic angina can be considered without significant epicardial coronary stenosis (Class IIb) [S75]  After DES implantation, no evidence of increased frequency of ACh-induced vasospasm with beta-blocker therapy [S76, 77]. | No study |
| **Vasodilating beta-blockers** | As for beta blockers  Vasodilatation via alpha blockade & NO generation | Carvedilol: Improvement in exercise tolerance, time to onset of angina, and 1mm ST-segment depression [S78]  Nebivolol: increased ischaemic and anginal thresholds [S79] | Suggested in AHA/ACC guidelines [S72]  *Results of NIRVANA Trial awaited* | As above | No study |
| **Calcium channel blockers** | Reduce heart rate and myocardial oxygen demand  Vasodilation via action on vascular smooth muscle  Reduce afterload | Class I recommendation [S71, 72] | Amlodipine: Improvement in exercise time (ChaMP-CMD) [S80] | Amlodipine reduces rate of angina episodes [S81]  Diltiazem – EDIT-CMD trial improves epicardial vasospasm on coronary function testing [S82] | No study |
| **Long-acting nitrates** | Vasodilation via action on smooth muscle  Reduce preload | Class IIa, Level of evidence B [S71] | Limited evidence  Limited benefit due to small vasodilatory effect on small resistance vessels [S103]. | Reduction in angina frequency [S104].  Did not improve long-term prognosis in patients when combined with CCBs [S105]. | No study |
| **Nicorandil** | Cytoprotective effects  Vasodilation through NO donation | IONA Trial [S83]  ESC 2024 Class IIb, level of evidence B [S71] | Limited evidence with small number of studies in CMD  [S84-86] | Limited evidence  Reduction in ergometrine-induced coronary spasm [S87] | No RCT  Limited evidence with small studies in RA [S88] |
| **Metabolic modifiers** |  |  |  |  |  |
| **Ranolazine** | Inhibition of late inward sodium current  Improves ionic homeostasis and myocardial energetics  Reduce myocardial oxygen demand | ESC 2024 Class IIa, level of evidence B recommendation [S71]  CARISA Trial [S89]  RIVER-PCI [S90] | Improvement in exercise time [S80]  Improvements in symptoms, quality of life, exercise performance and CFR [S91, 92]  Varying reports of effect on SAQ angina [S93, 94]  No improvement in symptoms or microvascular function (MARINA Trial) [S95] | No study | No RCT  Symptomatic improvement in observational cohort studies, no RCT  (Ranolazine Refractory Angina Registry) & Ling et al. [S96, 97] |
| **Trimetazidine** | Partial inhibition of β-oxidation & increases glucose oxidation  Increases cellular tolerance to ischaemia | ESC 2024 Class IIb, level of evidence B [S71]  AT-PCI trial [S98]  PATMOS trial [S99]  Meta-analysis of 13 studies showing clinical efficacy [S100] | Limited evidence  Improved total exercise time, time to 1mm ST-segment depression and maximum ST-segment depression [S101]  Another study failed to show benefit [S102] | No additional benefit on clinical outcomes when added to diltiazem and nitrates [S103] | No RCT  Meta-analysis showed improvements in walking time and angina severity in patients not suitable for revascularisation [S104] |
|  |  |  |  |  |  |
| **Ivabradine** | Reduce heart rate through inhibition of *I_f_* | ESC 2024 Class IIa, level of evidence B, for LVEF<40% and SR>70bpm [S71]  BEAUTIFUL & SIGNIFY Trials [S105, 106] | Improvement in SAQ [S94, 107]  No improvement in time to 1mm ST-segment depression or effect on microvascular function [S94] | No study | No study |
| **L-arginine** | Substrate for NO synthase  Improve endothelium-dependent vasodilation | Limited evidence [S108] | Limited evidence  Suggested improvements in endothelial function | Limited evidence  Long-term supplementation improved small-vessel endothelial function with improvement and symptoms [S109] | No study |
| **Rho-kinase inhibitors** | Coronary  vasodilation | Limited evidence  Improvements on treadmill exercise test observed [S110] | Limited evidence  Improvement in microvascular resistance in patients also with VSA [S111] | Improvement in ischaemia in microvascular spasm [S112, 113] | No study |
| **ACEi/ARB** | Inhibition of the effects of angiotensin II  Improves endothelial function | No study on its anti-anginal effect | CorMiCA [S114]  WISE Substudy [S115]  AHA/ACC 2023 Table 17 [S72] | Limited evidence  Suggested improvements in angina when combined with CCB [S116] | No study |
| ACEi: angiotensin-converting enzyme inhibitors; ACh: acetylcholine; AHA: American Heart Association; ACC: American College of Cardiology; bpm: beats per minute; ARB: angiotensin receptor blocker; CAD: coronary artery disease; CCB: calcium channel blocker; CFR: coronary flow reserve; cGMP: guanosine 3’, 5’-cyclic monophosphate; CMD: coronary microvascular dysfunction; DES: drug-eluting stent; ESC: European Society of Cardiology; ET:1: endothelin-1; I*_f_*: funny current; LVEF: left ventricular ejection fraction; NO: nitric oxide; RA: refractory angina; RCT: randomised controlled trial; SAQ: Seattle Angina Questionnaire; SR: sinus rhythm; VSA: vasospastic angina. | | | | | |

**Supplementary Figure 1. Advanced pain management approaches in patients with refractory angina. *Adapted from [S117, 118].***

AP: angina pectoris

BK: bradykinin

CCS: Canadian Cardiovascular Society

CNS: central nervous system

GABA: gamma-aminobutyric acid

SCS: spinal cord stimulation

SENS: subcutaneous electrical nerve stimulation

STT: spinothalamic tract

TENS: transcutaneous electrical nerve stimulation


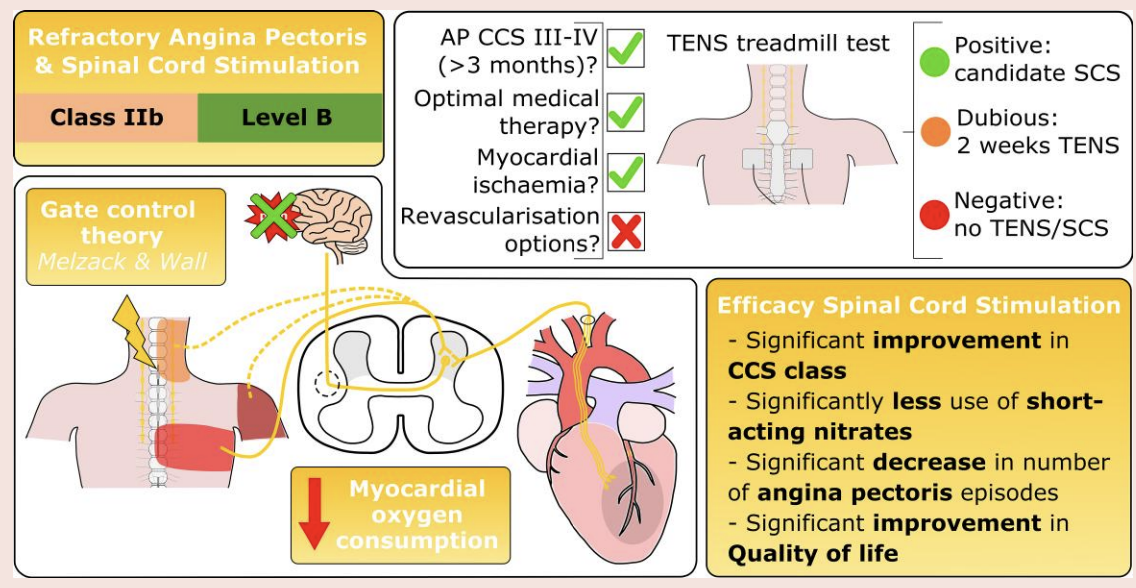

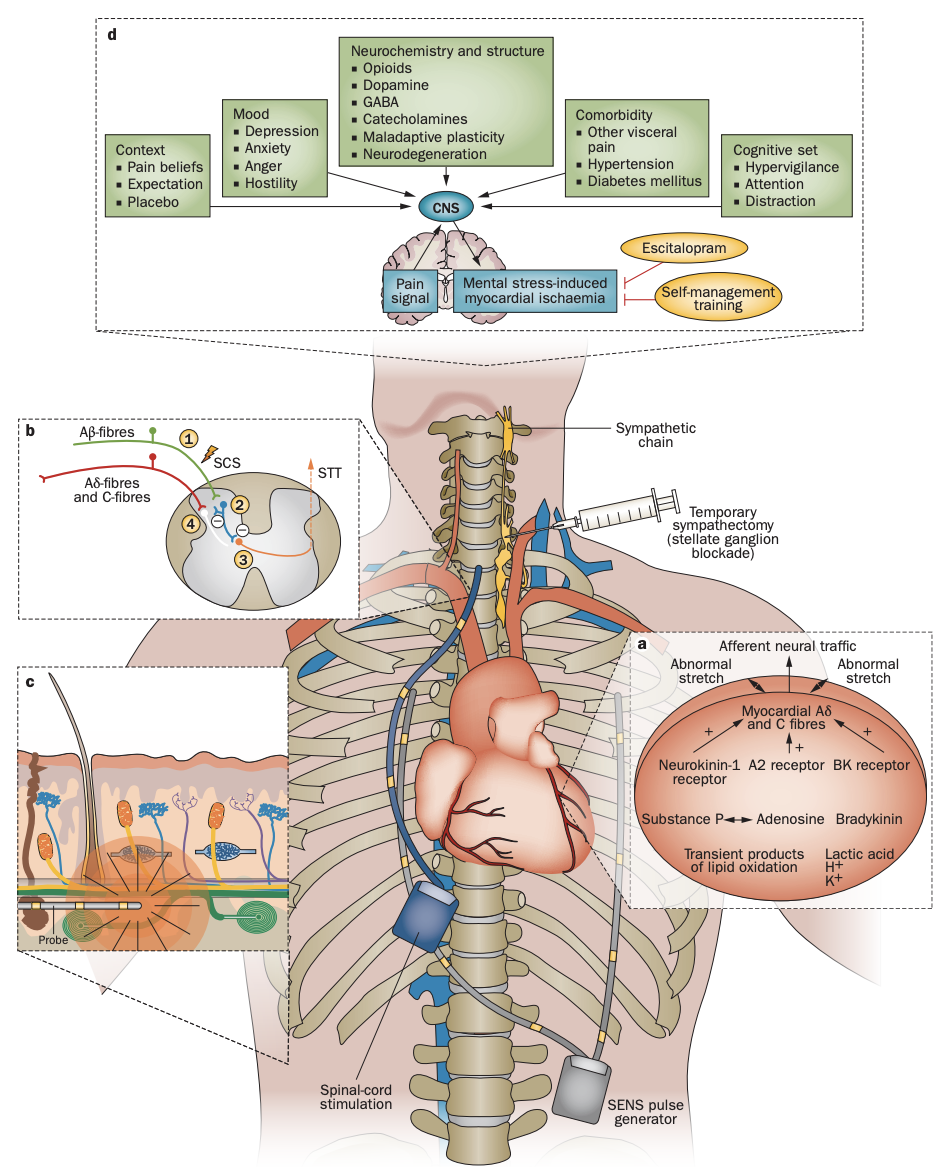


**Supplementary Table 1. Reported rates of persistent angina after successful revascularisation.**

| **Study** | **PCI/CABG** | **Follow-up** | **Rate of persistent angina (%)** |
| --- | --- | --- | --- |
| ACME [S119] | POBA | 6 months | 36 |
| ACME 2 [S120] | POBA | 6 months | 47 |
| CASS Registry [S121] | CABG | 1 year | 24 |
|  |  | 6 years | 40 |
| SOS [S122] | PCI | 1 year | 34 |
|  | CABG |  | 21 |
| ART [S123] | CABG | 1 year | 28-31 |
| MASS [S124] | PCI | 3 years | 2 |
|  | CABG |  | 18 |
| MASS 2 [S125] | PCI | 1 year | 48 |
|  | CABG |  | 41 |
| ARTS [S126] | PCI | 1 year | 21.1 |
|  | CABG |  | 10.5 |
| RITA-2 [S127] | PCI | 1-3 years | 30-40 |
| BARI-2D [S128] | PCI or CABG | 1 year | 60 |
|  |  | 5 years | 39 |
| COURAGE [S129] | PCI | 1 year | 34 |
|  |  | 3 years | 28 |
|  |  | 5 years | 26 |
| SYNTAX [S130] | PCI | 1 year | 28.4 |
|  | CABG |  | 23.7 |
| FREEDOM [S131] | PCI | 1 year | 20.5 |
|  | CABG |  | 16.5 |
| EXCEL [S132] | PCI | 1 year | 21.1 |
|  | CABG |  | 20.5 |
| SYNTAX Extend Survival Study [S133] | PCI | 1 year | 28.3 |
|  | CABG |  | 23.7 |
| ISCHEMIA [S134, 135] | PCI | 1 year | 66 |
| FAME-2 [S1] | PCI | 1 year | 5.9 |
|  |  | 3 years | 5.9 |
|  |  | 5 years | 7.4 |
| DEFINE-PCI [S136] | PCI | 1 year | 20.6 |
| ORBITA-2 [S137] | PCI | 12 weeks | 60.2 |
| CABG: coronary artery bypass grafting; PCI: percutaneous coronary intervention | | | |

**Supplementary Table 2. Non-coronary causes of chest pain (adapted from [S138-140]).**

| **Cardiac** | - Hypertensive heart disease - Left ventricular outflow tract obstruction - Hypertrophic cardiomyopathy - Dilated cardiomyopathy - Infiltrative cardiomyopathies - Left bundle branch block - Pericarditis - Aortic valve disease - Aortic dissection - Congenital cardiac abnormalities |
| --- | --- |
| **High output states** | - Anaemia - Thyrotoxicosis |
| **Gastro-intestinal** | - Gastro-oeseophageal reflux disease/gastritis/oesophagitis - Hiatus hernia - Oesophageal spasm - Peptic ulcer disease - Gallstones - Cholecystitis - Pancreatitis |
| **Musculo-skeletal/chest wall** | - Post-sternotomy pain syndrome - Sternal wire fracture - Costochondritis - Intercostal neuralgia |
| **Respiratory** | - Pulmonary embolism - Pleural irritation - Pulmonary hypertension - Pneumothorax/haemothorax - Pneumomediastinum - Pneumonia - Bronchitis - Malignancy |

**Supplementary Table 3. Investigational drugs for refractory angina.**

| **Drug** | **Mechanism of action** | **Evidence of efficacy** | | | |
| --- | --- | --- | --- | --- | --- |
|  |  | **Stable angina/obstructive epicardial CAD** | **Coronary microvascular dysfunction** | **Vasospasm** | **Refractory angina** |
| **Molsidomine** | Vasodilation through NO donation | Reduced incidence of anginal attacks and use of nitrates [S141] | No study | Limited evidence  Potentially as effective as nifedipine in reducing ergometrine-induced spasm [S142]. | No study |
| **Metabolic modifiers** |  |  |  |  |  |
| **Perhexiline** | Inhibition of fatty acid oxidation | Limited evidence [S143] | No evidence suggesting benefit | Limited evidence  No improvement in adverse events compared to nifedipine, diltiazem or verapamil [S144] | Symptomatic improvement in a small cohort study [S145]  Retrospective data suggesting improvement in symptoms in heart failure and RA [S146] |
| **Allopurinol** | Decreases myocardial oxygen demand | Increase in time to ST depression, total exercise time and time to chest pain [S147] | No difference in maximum exercise time or CFR [S148] | No study | No study |
| **Mildronate** | Reduces fatty acid oxidation | Limited evidence  Improvement in total exercise time [S149] | No study | No study | No study |
|  |  |  |  |  |  |
| **Soluble guanylate cyclase stimulants** | Vasodilation through stimulation of cGMP | No study | No study | No study | No study |
| **Statins** | Inhibition of HMG-CoA reductase  Lipid lowering  Anti-inflammatory  Improves endothelial function | Reductions in angina frequency and hospitalisation (TNT, PROVE-IT, 4S) [S150-152] | Limited evidence  Small studies suggest improvements in CFR [S153] | Limited evidence  Improved endothelial function. May reduce frequency of coronary vasospasm in addition to CCB [S154] | No study |
| **SGLT-2 inhibitors** | Inhibition of sodium-glucose cotransporters on proximal convoluted renal tubules  Increase in natriuresis and glucose excretion | No study on symptom improvement | Limited evidence  No improvement in CFVR in patients with T2DM after empagliflozin [S155] | No study | Early study of benefit in RA (EMPT-ANGINA) [S156] |
| **Denopamine** | Combined beta-1 adrenergic agonist and alpha-receptor blockade | No study | No study | Limited evidence – can be considered in patients without obstructive epicardial CAD and vasospasm confirmed by ACh provocation [S77] | No study |
| **Cilostazol** | Inhibits PDE-3  Vasodilatation through smooth muscle relaxation and improved endothelial function  Inhibits platelet aggregation | Limited evidence  Suggested to reduce risk of angina [S157] | No study | Limited evidence – trials are small and of short duration of therapy  May improve coronary vasoreactivity [S158], frequency and duration of angina [S159, 160] | No study |
| **PDE-5 inhibitors** | Vasodilation through effect on cGMP | Limited data. Improvement in time to angina and total exercise time [S161] | No data on clinical efficacy  Improvements in CFR in a small study [S162] | No study | No study |
| **ET-1 antagonists** | Coronary vasodilation | No study | No evidence of clinical efficacy [S163] | No evidence of clinical efficacy [S164] | No study |
| ACh: acetylcholine; AHA: American Heart Association; ACC: American College of Cardiology; CAD: coronary artery disease; CCB: calcium channel blocker; CFR: coronary flow reserve; cGMP: guanosine 3’, 5’-cyclic monophosphate; CMD: coronary microvascular dysfunction; NO: nitric oxide; PDE-3: phosphodiesterase-3; PDE-5: phosphodiesterase-5; RA: refractory angina; SGLT-2: sodium-glucose cotransporter-2; T2DM: type 2 diabetes mellitus. | | | | | |

**Supplementary Table 4. Non-pharmacological options for anti-ischaemic therapy.**

| **Anti-ischaemic therapy** | **Description** | **Proposed mechanism of action** | **Randomised Study** | **Guideline recommendation** |
| --- | --- | --- | --- | --- |
| Coronary sinus reducer (CSR) | Balloon-mounted device percutaneously inserted into the coronary sinus | Reduction in microvascular resistance [S165]  Redistribution of myocardial perfusion [S166-171]  Improvement in endo:epi perfusion ratio [S167, 171] | COSIRA [S172]  ORBITA-COSMIC [S171] | IIb [S71] |
| Enhanced external counterpulsation (EECP) | Sequential inflation of a series of compressive cuffs on the lower limbs | Creates an effect similar to a balloon pump promoting retrograde aortic flow to increase diastolic pressure, improve coronary perfusion, venous return and cardiac output | MUST-EECP [S173]  *(stable angina)* | - |
| Biologics | Cell therapy (CD34+/CD133+) | Proangiogenic paracrine effects | Pooled analysis of ACT-34, ACT-34 extension & RENEW [S174]  REGENT-VSEL [S175]  Meta-analyses of RCTs [S176, 177]  FREEDOM [S178]  IMPROvE-CED [S179] |  |
|  | Gene transfer therapy  Viral vectors  Plasmids | Promotion of neovascularisation (e.g. through expression of VEGF) | Euroinject [S180]  AGENT-3 & AGENT-4 [S181]  KAT301 [S182]  EXACT [S183] | - |
| Extracorporeal shockwave therapy (ECSWT) | Delivery of low-energy shockwaves to the border zones of ischaemic myocardium | Reduction in ischaemia through induction of local vasodilation and neovascularisation | Several small RCTs [S184-186] | - |
| Low-intensity pulse ultrasound (LIPUS) | Delivery of pulsed ultrasound | Induction of mechano-transduction to promote neovascularisation and upregulate endothelial NO synthase [S162] | Shindo et al. [S187] | - |
| RCT: randomised controlled trial; NO: nitric oxide; VEGF: vascular endothelial growth factor | | | | |

**Supplementary Table 5.** **Pharmacological and non-pharmacological options for advanced pain management in refractory angina.**

| 1. **Pharmacology** | | | | | | | | |
| --- | --- | --- | --- | --- | --- | --- | --- | --- |
| **Drug** | **Mechanism of Action** | | | | **Evidence** | | **Patient groups** | |
| Tricyclic antidepressants (Imipramine) | Modulatory effects on norepinephrine uptake and anticholinergic effect | | | | 52% ↓ in chest pain episodes [S188].  No improvement in quality of life [S189]. | | Unobstructed coronary arteries | |
| SSRI (Escitalopram) | Inhibition of the reuptake of serotonin into the presynaptic neuron | | | | No improvement in exercise-induced ischaemia [S190] | | CAD | |
| SNRI (duloxetine, venlafaxine) | Inhibition of the reuptake of serotonin and norepinephrine into the presynaptic neuron | | | | Nil | |  | |
| Gabapentinoids (pregabalin) | Bind to voltage-gated calcium channels and reduce the synaptic release of several neurotransmitters | | | | Nil | |  | |
| Opiates | μ-opioid receptor agonist and effects descending inhibitory pathways modulating nociception | | | | Nil | |  | |
| 1. **Neuromodulation** | | | | | | | | |
| **Modality** | | **Placebo-controlled study** | **Participants (N)** | **Primary outcome** | | **Result** | | **p-value/95%-CI** |
| **Transcutaneous electrical nerve stimulation (TENS)** | | No placebo-controlled studies available.  Case series: Nitz et al. (1993) [S191], West et al. (1993) [S192], Meyler et al. (1994) [S193] | | | | | | |
| **Subcutaneous electrical nerve stimulation (SENS)** | | No placebo-controlled studies available. Case series: Buiten al. (2011) [S194], Goroszeniuk et al. (2012) [S195] | | | | | | |
| **Spinal cord stimulation (SCS)** | | Eddicks et al (2007) [S196]  Lanza et al (2011) [S197]  Zipes et al (2012) [S198]  Eldabe et al (2016) [S199] | 12  25  68  29 | Walking distance  Angina attacks (week)  Daily anginal attacks  Angina frequency (SAQ) | | + 57m  -12 episodes  -1.19 vs -1.29 episodes  -12.8 points | | p = 0.013  p = 0.002  p = 0.445  95%-CI = -29.1 – 3.5 |
| **Sympathectomy** | | Denby et al (2015) [S200] | 51 | Angina frequency (week) | | -31% vs. -31% | | p = NS |

| 1. Cognitive behavioural therapy and cardiac rehabilitation interventions (Adapted from [S177]) | | | | | | |
| --- | --- | --- | --- | --- | --- | --- |
| Author & Year | **Design** | **Setting** | **Participants** | **Intervention** | **Follow up** | **Outcomes** |
| Asbury et al. (2012) [S201] | RCT | Cardiology (including specialist RA clinic) and pain clinic outpatients | 40  1 hospitalised.  Final analysis: 19 intervention; 20 control | 8-week CR and symptom monitoring programme.  Control: 8-week symptom monitoring only | Assessed at baseline, after final session and 8 weeks after intervention | Improved physical ability and health anxiety |
| Mittal et al. (2022) [S202] | RCT | Cardiology (persistent non-cardiac chest pain in a rapid access chest pain clinic) | 33  27 completed the study | 2-hour, weekly, online guided 8-week course of mindfulness-based cognitive therapy | Assessed at baseline and after 8-week period | Improvement in general anxiety and mindfulness, and a trend towards improvement in SAQ scores |
| Moore et al. (2005) [S203] | Prospective case series | Specialist RA clinic at a tertiary cardiac referral centre | 69  2 died, 1 lost to follow-up | RA programme consisting of education, drug optimisation, CR and CBT | Assessed at baseline and one year after intervention | Improved health-related quality of life measured by SAQ and SF-12 |
| Moore et al. (2007) [S204] | Retrospective case series | Specialist RA clinic at a tertiary cardiac referral centre | 433 | Two-hour interview, stress management advice, relaxation training and a graduated exercise program | Data was collected for one year pre- and post-intervention | Immediate and sustained reduction in hospitalisation rate and lower mortality compared to surgical revascularisation |
| Patel et al. (2016) [S205] | Prospective case series | Specialised RA clinic | 33  3 underwent revascularisation and were excluded | Four-week 'pragmatic rehabilitation course’ combining CBT and an education programme | Assessed at baseline, one-month and two years | Improved quality of life (measured via SF-36) and mood (HADS). No change in angina frequency or GTN usage |
| Tinson et al. (2016) [S206] | Retrospective case series | Outpatients, referred by cardiologists and primary care physicians | 135  Missing data for 66 participants but still included in some analyses | 9-week angina management programme run by a cognitive-behavioural therapist and physiotherapist | Assessed at baseline, after final session and two months after intervention | Reduction in self-reported angina frequency, duration, and GTN usage. Improvements in quality of life/disability, angina misconceptions, self-efficacy and mood. |
| CAD: coronary artery disease; CBT: cognitive behavioural therapy; CI: confidence interval; CR: cardiac rehabilitation; GTN: glyceryl trinitrate; HADS: Hospital Anxiety and Depression scale; RA: refractory angina; RCT: randomised controlled trial; SAQ: Seattle Angina Questionnaire; SF-12: Short Form-12 Health Survey; SF-36: Short Form-36 Health Survey; SSRI: selective serotonin reuptake inhibitors; SNRI: serotonin and norepinephrine reuptake inhibitors. | | | | | | |

**References**

S1. Xaplanteris P, Fournier S, Pijls NHJ, Fearon WF, Barbato E, Tonino PAL, et al. Five-Year Outcomes with PCI Guided by Fractional Flow Reserve. N Engl J Med. 2018;379(3):250-9.

S2. Ferrari M, Schnell B, Werner GS, Figulla HR. Safety of deferring angioplasty in patients with normal coronary flow velocity reserve. J Am Coll Cardiol. 1999;33(1):82-7.

S3. Everaars H, de Waard GA, Driessen RS, Danad I, van de Ven PM, Raijmakers PG, et al. Doppler Flow Velocity and Thermodilution to Assess Coronary Flow Reserve: A Head-to-Head Comparison With [JACC Cardiovasc Interv. 2018;11(20):2044-54.

S4. Meuwissen M, Siebes M, Chamuleau SA, van Eck-Smit BL, Koch KT, de Winter RJ, et al. Hyperemic stenosis resistance index for evaluation of functional coronary lesion severity. Circulation. 2002;106(4):441-6.

S5. Collet C, Munhoz D, Mizukami T, Sonck J, Matsuo H, Shinke T, et al. Influence of Pathophysiological Patterns of Coronary Artery Disease on Immediate Percutaneous Coronary Intervention Outcomes. Circulation. 2024.

S6. Escaned J, Berry C, De Bruyne B, Shabbir A, Collet C, Lee JM, et al. Applied coronary physiology for planning and guidance of percutaneous coronary interventions. A clinical consensus statement from the European Association of Percutaneous Cardiovascular Interventions (EAPCI) of the European Society of Cardiology. EuroIntervention. 2023;19(6):464-81.

S7. van de Hoef TP, Lee JM, Echavarria-Pinto M, Koo BK, Matsuo H, Patel MR, et al. Non-hyperaemic coronary pressure measurements to guide coronary interventions. Nat Rev Cardiol. 2020;17(10):629-40.

S8. Kogame N, Ono M, Kawashima H, Tomaniak M, Hara H, Leipsic J, et al. The Impact of Coronary Physiology on Contemporary Clinical Decision Making. JACC Cardiovasc Interv. 2020;13(14):1617-38.

S9. Tu S, Westra J, Yang J, von Birgelen C, Ferrara A, Pellicano M, et al. Diagnostic Accuracy of Fast Computational Approaches to Derive Fractional Flow Reserve From Diagnostic Coronary Angiography: The International Multicenter FAVOR Pilot Study. JACC Cardiovasc Interv. 2016;9(19):2024-35.

S10. Westra J, Andersen BK, Campo G, Matsuo H, Koltowski L, Eftekhari A, et al. Diagnostic Performance of In-Procedure Angiography-Derived Quantitative Flow Reserve Compared to Pressure-Derived Fractional Flow Reserve: The FAVOR II Europe-Japan Study. J Am Heart Assoc. 2018;7(14).

S11. Xu B, Tu S, Qiao S, Qu X, Chen Y, Yang J, et al. Diagnostic Accuracy of Angiography-Based Quantitative Flow Ratio Measurements for Online Assessment of Coronary Stenosis. J Am Coll Cardiol. 2017;70(25):3077-87.

S12. Masdjedi K, van Zandvoort LJC, Balbi MM, Gijsen FJH, Ligthart JMR, Rutten MCM, et al. Validation of a three-dimensional quantitative coronary angiography-based software to calculate fractional flow reserve: the FAST study. EuroIntervention. 2020;16(7):591-9.

S13. Li J, Gong Y, Wang W, Yang Q, Liu B, Lu Y, et al. Accuracy of computational pressure-fluid dynamics applied to coronary angiography to derive fractional flow reserve: FLASH FFR. Cardiovasc Res. 2020;116(7):1349-56.

S14. Fearon WF, Achenbach S, Engstrom T, Assali A, Shlofmitz R, Jeremias A, et al. Accuracy of Fractional Flow Reserve Derived From Coronary Angiography. Circulation. 2019;139(4):477-84.

S15. Gibbons RJ, Balady GJ, Bricker JT, Chaitman BR, Fletcher GF, Froelicher VF, et al. ACC/AHA 2002 guideline update for exercise testing: summary article. A report of the American College of Cardiology/American Heart Association Task Force on Practice Guidelines (Committee to Update the 1997 Exercise Testing Guidelines). J Am Coll Cardiol. 2002;40(8):1531-40.

S16. Belardinelli R, Lacalaprice F, Carle F, Minnucci A, Cianci G, Perna G, et al. Exercise-induced myocardial ischaemia detected by cardiopulmonary exercise testing. Eur Heart J. 2003;24(14):1304-13.

S17. Munhoz EC, Hollanda R, Vargas JP, Silveira CW, Lemos AL, Hollanda RM, et al. Flattening of oxygen pulse during exercise may detect extensive myocardial ischemia. Med Sci Sports Exerc. 2007;39(8):1221-6.

S18. Chaudhry S, Arena R, Wasserman K, Hansen JE, Lewis GD, Myers J, et al. Exercise-induced myocardial ischemia detected by cardiopulmonary exercise testing. Am J Cardiol. 2009;103(5):615-9.

S19. Dominguez-Rodriguez A, Abreu-Gonzalez P, Gomez MA, Garcia-Baute MdC, Arroyo-Ucar E, Avanzas P, et al. Myocardial perfusion defects detected by cardiopulmonary exercise testing: Role of VE/VCO2 slope in patients with chest pain suspected of coronary artery disease. International journal of cardiology. 2012;155(3):470-1.

S20. Popovic D, Guazzi M, Jakovljevic DG, Lasica R, Banovic M, Ostojic M, et al. Quantification of coronary artery disease using different modalities of cardiopulmonary exercise testing. Int J Cardiol. 2019;285:11-3.

S21. Ganesananthan S, Rajkumar CA, Foley M, Thompson D, Nowbar AN, Seligman H, et al. Cardiopulmonary exercise testing and efficacy of percutaneous coronary intervention: a substudy of the ORBITA trial. Eur Heart J. 2022;43(33):3132-45.

S22. Min JK, Taylor CA, Achenbach S, Koo BK, Leipsic J, Nørgaard BL, et al. Noninvasive Fractional Flow Reserve Derived From Coronary CT Angiography: Clinical Data and Scientific Principles. JACC Cardiovasc Imaging. 2015;8(10):1209-22.

S23. Rossi A, Dharampal A, Wragg A, Davies LC, van Geuns RJ, Anagnostopoulos C, et al. Diagnostic performance of hyperaemic myocardial blood flow index obtained by dynamic computed tomography: does it predict functionally significant coronary lesions? Eur Heart J Cardiovasc Imaging. 2014;15(1):85-94.

S24. Pellikka PA, Arruda-Olson A, Chaudhry FA, Chen MH, Marshall JE, Porter TR, et al. Guidelines for Performance, Interpretation, and Application of Stress Echocardiography in Ischemic Heart Disease: From the American Society of Echocardiography. J Am Soc Echocardiogr. 2020;33(1):1-41.e8.

S25. Rigo F, Sicari R, Gherardi S, Djordjevic-Dikic A, Cortigiani L, Picano E. The additive prognostic value of wall motion abnormalities and coronary flow reserve during dipyridamole stress echo. Eur Heart J. 2008;29(1):79-88.

S26. Porter TR, Adolphson M, High RR, Smith LM, Olson J, Erdkamp M, et al. Rapid detection of coronary artery stenoses with real-time perfusion echocardiography during regadenoson stress. Circ Cardiovasc Imaging. 2011;4(6):628-35.

S27. Dorbala S, Ananthasubramaniam K, Armstrong IS, Chareonthaitawee P, DePuey EG, Einstein AJ, et al. Single Photon Emission Computed Tomography (SPECT) Myocardial Perfusion Imaging Guidelines: Instrumentation, Acquisition, Processing, and Interpretation. J Nucl Cardiol. 2018;25(5):1784-846.

S28. Wang J, Li S, Chen W, Chen Y, Pang Z, Li J. Diagnostic efficiency of quantification of myocardial blood flow and coronary flow reserve with CZT dynamic SPECT imaging for patients with suspected coronary artery disease: a comparative study with traditional semi-quantitative evaluation. Cardiovasc Diagn Ther. 2021;11(1):56-67.

S29. Wang L, Zheng Y, Zhang J, Wang M, Wu D, Wang Y, et al. Diagnostic value of quantitative myocardial blood flow assessment by NaI(Tl) SPECT in detecting significant stenosis: a prospective, multi-center study. J Nucl Cardiol. 2023;30(2):769-80.

S30. Bateman TM, Heller GV, Beanlands R, Calnon DA, Case J, deKemp R, et al. Practical Guide for Interpreting and Reporting Cardiac PET Measurements of Myocardial Blood Flow: An Information Statement from the American Society of Nuclear Cardiology, and the Society of Nuclear Medicine and Molecular Imaging. J Nucl Med. 2021;62(11):1599-615.

S31. Hundley WG, Bluemke DA, Bogaert J, Flamm SD, Fontana M, Friedrich MG, et al. Society for Cardiovascular Magnetic Resonance (SCMR) guidelines for reporting cardiovascular magnetic resonance examinations. J Cardiovasc Magn Reson. 2022;24(1):29.

S32. Nagel E, Klein C, Paetsch I, Hettwer S, Schnackenburg B, Wegscheider K, et al. Magnetic resonance perfusion measurements for the noninvasive detection of coronary artery disease. Circulation. 2003;108(4):432-7.

S33. Kotecha T, Martinez-Naharro A, Boldrini M, Knight D, Hawkins P, Kalra S, et al. Automated Pixel-Wise Quantitative Myocardial Perfusion Mapping by CMR to Detect Obstructive Coronary Artery Disease and Coronary Microvascular Dysfunction: Validation Against Invasive Coronary Physiology. JACC Cardiovasc Imaging. 2019;12(10):1958-69.

S34. Wang S, Kim P, Wang H, Ng MY, Arai AE, Singh A, et al. Myocardial Blood Flow Quantification Using Stress Cardiac Magnetic Resonance Improves Detection of Coronary Artery Disease. JACC Cardiovasc Imaging. 2024.

S35. Perera D, Berry C, Hoole SP, Sinha A, Rahman H, Morris PD, et al. Invasive coronary physiology in patients with angina and non-obstructive coronary artery disease: a consensus document from the coronary microvascular dysfunction workstream of the British Heart Foundation/National Institute for Health Research Partnership. Heart. 2022.

S36. Demir OM, Rahman H, van de Hoef TP, Escaned J, Piek JJ, Plein S, et al. Invasive and non-invasive assessment of ischaemia in chronic coronary syndromes: translating pathophysiology to clinical practice. Eur Heart J. 2022;43(2):105-17.

S37. Ong P, Camici PG, Beltrame JF, Crea F, Shimokawa H, Sechtem U, et al. International standardization of diagnostic criteria for microvascular angina. Int J Cardiol. 2018;250:16-20.

S38. Patel N, Petraco R, Dall'Armellina E, Kassimis G, De Maria GL, Dawkins S, et al. Zero-Flow Pressure Measured Immediately After Primary Percutaneous Coronary Intervention for ST-Segment Elevation Myocardial Infarction Provides the Best Invasive Index for Predicting the Extent of Myocardial Infarction at 6 Months: An OxAMI Study (Oxford Acute Myocardial Infarction). JACC Cardiovasc Interv. 2015;8(11):1410-21.

S39. Rahman H, Demir OM, Ryan M, McConkey H, Scannell C, Ellis H, et al. Optimal Use of Vasodilators for Diagnosis of Microvascular Angina in the Cardiac Catheterization Laboratory. Circ Cardiovasc Interv. 2020;13(6):e009019.

S40. Pijls NH, De Bruyne B, Smith L, Aarnoudse W, Barbato E, Bartunek J, et al. Coronary thermodilution to assess flow reserve: validation in humans. Circulation. 2002;105(21):2482-6.

S41. Fearon WF, Kobayashi Y. Invasive Assessment of the Coronary Microvasculature: The Index of Microcirculatory Resistance. Circ Cardiovasc Interv. 2017;10(12).

S42. Scarsini R, De Maria GL, Borlotti A, Kotronias RA, Langrish JP, Lucking AJ, et al. Incremental Value of Coronary Microcirculation Resistive Reserve Ratio in Predicting the Extent of Myocardial Infarction in Patients with STEMI. Insights from the Oxford Acute Myocardial Infarction (OxAMI) Study. Cardiovasc Revasc Med. 2019;20(12):1148-55.

S43. Boerhout CKM, Lee JM, de Waard GA, Mejia-Renteria H, Lee SH, Jung JH, et al. Microvascular resistance reserve: diagnostic and prognostic performance in the ILIAS registry. Eur Heart J. 2023;44(30):2862-9.

S44. Gallinoro E, Bertolone DT, Mizukami T, Paolisso P, Bermpeis K, Munhoz D, et al. Continuous vs Bolus Thermodilution to Assess Microvascular Resistance Reserve. JACC Cardiovasc Interv. 2023;16(22):2767-77.

S45. Xaplanteris P, Fournier S, Keulards DCJ, Adjedj J, Ciccarelli G, Milkas A, et al. Catheter-Based Measurements of Absolute Coronary Blood Flow and Microvascular Resistance: Feasibility, Safety, and Reproducibility in Humans. Circ Cardiovasc Interv. 2018;11(3):e006194.

S46. Belmonte M, Gallinoro E, Pijls NHJ, Bertolone DT, Keulards DCJ, Viscusi MM, et al. Measuring Absolute Coronary Flow and Microvascular Resistance by Thermodilution: JACC Review Topic of the Week. J Am Coll Cardiol. 2024;83(6):699-709.

S47. Everaars H, de Waard GA, Schumacher SP, Zimmermann FM, Bom MJ, van de Ven PM, et al. Continuous thermodilution to assess absolute flow and microvascular resistance: validation in humans using [15O]H2O positron emission tomography. Eur Heart J. 2019;40(28):2350-9.

S48. Gallinoro E, Bertolone DT, Fernandez-Peregrina E, Paolisso P, Bermpeis K, Esposito G, et al. Reproducibility of bolus versus continuous thermodilution for assessment of coronary microvascular function in patients with ANOCA. EuroIntervention. 2023;19(2):e155-e66.

S49. De Bruyne B, Pijls NHJ, Gallinoro E, Candreva A, Fournier S, Keulards DCJ, et al. Microvascular Resistance Reserve for Assessment of Coronary Microvascular Function: JACC Technology Corner. J Am Coll Cardiol. 2021;78(15):1541-9.

S50. Jansen TPJ, de Vos A, Paradies V, Dimitriu-Leen A, Crooijmans C, Elias-Smale S, et al. Continuous Versus Bolus Thermodilution-Derived Coronary Flow Reserve and Microvascular Resistance Reserve and Their Association With Angina and Quality of Life in Patients With Angina and Nonobstructive Coronaries: A Head-to-Head Comparison. J Am Heart Assoc. 2023;12(16):e030480.

S51. Mejía-Rentería H, Wang L, Chipayo-Gonzales D, van de Hoef TP, Travieso A, Espejo C, et al. Angiography-derived assessment of coronary microcirculatory resistance in patients with suspected myocardial ischaemia and non-obstructive coronary arteries. EuroIntervention. 2023;18(16):e1348-e56.

S52. Scarsini R, Shanmuganathan M, Kotronias RA, Terentes-Printzios D, Borlotti A, Langrish JP, et al. Angiography-derived index of microcirculatory resistance (IMR. Int J Cardiovasc Imaging. 2021;37(6):1801-13.

S53. Sternheim D, Power DA, Samtani R, Kini A, Fuster V, Sharma S. Myocardial Bridging: Diagnosis, Functional Assessment, and Management: JACC State-of-the-Art Review. J Am Coll Cardiol. 2021;78(22):2196-212.

S54. Beltrame JF, Crea F, Kaski JC, Ogawa H, Ong P, Sechtem U, et al. International standardization of diagnostic criteria for vasospastic angina. Eur Heart J. 2017;38(33):2565-8.

S55. Sinha A, Dutta U, Demir OM, De Silva K, Ellis H, Belford S, et al. Rethinking False Positive Exercise Electrocardiographic Stress Tests by Assessing Coronary Microvascular Function. J Am Coll Cardiol. 2024;83(2):291-9.

S56. Bechsgaard DF, Hove JD, Suhrs HE, Bové KB, Shahriari P, Gustafsson I, et al. Women with coronary microvascular dysfunction and no obstructive coronary artery disease have reduced exercise capacity. Int J Cardiol. 2019;293:1-9.

S57. Pargaonkar VS, Kobayashi Y, Kimura T, Schnittger I, Chow EKH, Froelicher VF, et al. Accuracy of non-invasive stress testing in women and men with angina in the absence of obstructive coronary artery disease. Int J Cardiol. 2019;282:7-15.

S58. Schroder J, Michelsen MM, Mygind ND, Suhrs HE, Bove KB, Bechsgaard DF, et al. Coronary flow velocity reserve predicts adverse prognosis in women with angina and no obstructive coronary artery disease: results from the iPOWER study. Eur Heart J. 2021;42(3):228-39.

S59. Barton D, Xie F, O'Leary E, Chatzizisis YS, Pavlides G, Porter TR. The Relationship of Capillary Blood Flow Assessments with Real Time Myocardial Perfusion Echocardiography to Invasively Derived Microvascular and Epicardial Assessments. J Am Soc Echocardiogr. 2019;32(9):1095-101.

S60. Taqui S, Ferencik M, Davidson BP, Belcik JT, Moccetti F, Layoun M, et al. Coronary Microvascular Dysfunction by Myocardial Contrast Echocardiography in Nonelderly Patients Referred for Computed Tomographic Coronary Angiography. J Am Soc Echocardiogr. 2019;32(7):817-25.

S61. Schindler TH, Fearon WF, Pelletier-Galarneau M, Ambrosio G, Sechtem U, Ruddy TD, et al. Myocardial Perfusion PET for the Detection and Reporting of Coronary Microvascular Dysfunction: A JACC: Cardiovascular Imaging Expert Panel Statement. JACC Cardiovasc Imaging. 2023;16(4):536-48.

S62. Murthy VL, Bateman TM, Beanlands RS, Berman DS, Borges-Neto S, Chareonthaitawee P, et al. Clinical Quantification of Myocardial Blood Flow Using PET: Joint Position Paper of the SNMMI Cardiovascular Council and the ASNC. J Nucl Med. 2018;59(2):273-93.

S63. Panting JR, Gatehouse PD, Yang GZ, Grothues F, Firmin DN, Collins P, et al. Abnormal subendocardial perfusion in cardiac syndrome X detected by cardiovascular magnetic resonance imaging. N Engl J Med. 2002;346(25):1948-53.

S64. Thomson LE, Wei J, Agarwal M, Haft-Baradaran A, Shufelt C, Mehta PK, et al. Cardiac magnetic resonance myocardial perfusion reserve index is reduced in women with coronary microvascular dysfunction. A National Heart, Lung, and Blood Institute-sponsored study from the Women's Ischemia Syndrome Evaluation. Circ Cardiovasc Imaging. 2015;8(4).

S65. Rahman H, Scannell CM, Demir OM, Ryan M, McConkey H, Ellis H, et al. High-Resolution Cardiac Magnetic Resonance Imaging Techniques for the Identification of Coronary Microvascular Dysfunction. JACC Cardiovasc Imaging. 2020.

S66. Lin S, Tremmel JA, Yamada R, Rogers IS, Yong CM, Turcott R, et al. A novel stress echocardiography pattern for myocardial bridge with invasive structural and hemodynamic correlation. J Am Heart Assoc. 2013;2(2):e000097.

S67. Pargaonkar VS, Rogers IS, Su J, Forsdahl SH, Kameda R, Schreiber D, et al. Accuracy of a novel stress echocardiography pattern for myocardial bridging in patients with angina and no obstructive coronary artery disease - A retrospective and prospective cohort study. Int J Cardiol. 2020;311:107-13.

S68. Forsdahl SH, Rogers IS, Schnittger I, Tanaka S, Kimura T, Pargaonkar VS, et al. Myocardial Bridges on Coronary Computed Tomography Angiography　- Correlation With Intravascular Ultrasound and Fractional Flow Reserve. Circ J. 2017;81(12):1894-900.

S69. Feldman RL, Hill JA, Whittle JL, Conti CR, Pepine CJ. Electrocardiographic changes with coronary artery spasm. Am Heart J. 1983;106(6):1288-97.

S70. Onaka H, Hirota Y, Shimada S, Kita Y, Sakai Y, Kawakami Y, et al. Clinical observation of spontaneous anginal attacks and multivessel spasm in variant angina pectoris with normal coronary arteries: evaluation by 24-hour 12-lead electrocardiography with computer analysis. J Am Coll Cardiol. 1996;27(1):38-44.

S71. Vrints C, Andreotti F, Koskinas KC, Rossello X, Adamo M, Ainslie J, et al. 2024 ESC Guidelines for the management of chronic coronary syndromes: Developed by the task force for the management of chronic coronary syndromes of the European Society of Cardiology (ESC) Endorsed by the European Association for Cardio-Thoracic Surgery (EACTS). European Heart Journal. 2024.

S72. Virani SS, Newby LK, Arnold SV, Bittner V, Brewer LC, Demeter SH, et al. 2023 AHA/ACC/ACCP/ASPC/NLA/PCNA Guideline for the Management of Patients With Chronic Coronary Disease: A Report of the American Heart Association/American College of Cardiology Joint Committee on Clinical Practice Guidelines. Circulation. 2023;148(9):e9-e119.

S73. Crea F, Pupita G, Galassi AR, el-Tamimi H, Kaski JC, Davies GJ, et al. Effects of theophylline, atenolol and their combination on myocardial ischemia in stable angina pectoris. Am J Cardiol. 1990;66(17):1157-62.

S74. Lanza GA, Colonna G, Pasceri V, Maseri A. Atenolol versus amlodipine versus isosorbide-5-mononitrate on anginal symptoms in syndrome X. Am J Cardiol. 1999;84(7):854-6, A8.

S75. Group JJW. Guidelines for diagnosis and treatment of patients with vasospastic angina (Coronary Spastic Angina) (JCS 2013). Circ J. 2014;78(11):2779-801.

S76. Sawano M, Katsuki T, Kitai T, Tamita K, Obunai K, Ikegami Y, et al. Beta blockers versus calcium channel blockers for provocation of vasospastic angina after drug-eluting stent implantation: a multicentre prospective randomised trial. Open Heart. 2020;7(2).

S77. Hokimoto S, Kaikita K, Yasuda S, Tsujita K, Ishihara M, Matoba T, et al. JCS/CVIT/JCC 2023 Guideline Focused Update on Diagnosis and Treatment of Vasospastic Angina (Coronary Spastic Angina) and Coronary Microvascular Dysfunction. Circ J. 2023;87(6):879-936.

S78. van der Does R, Eberhardt R, Derr I, Ehmer B, Rudorf J, Uberbacher HJ. Treatment of chronic stable angina with carvedilol in comparison with nifedipine s.r. Eur Heart J. 1991;12(1):60-4.

S79. Ruf G, Trenk D, Jähnchen E, Roskamm H. Determination of the anti-ischemic activity of nebivolol in comparison with atenolol. Int J Cardiol. 1994;43(3):279-85.

S80. Sinha A, Rahman H, Douiri A, Demir OM, De Silva K, Clapp B, et al. ChaMP-CMD: A Phenotype-Blinded, Randomized Controlled, Cross-Over Trial. Circulation. 2024;149(1):36-47.

S81. Chahine RA, Feldman RL, Giles TD, Nicod P, Raizner AE, Weiss RJ, et al. Randomized placebo-controlled trial of amlodipine in vasospastic angina. Amlodipine Study 160 Group. J Am Coll Cardiol. 1993;21(6):1365-70.

S82. Jansen TPJ, Konst RE, de Vos A, Paradies V, Teerenstra S, van den Oord SCH, et al. Efficacy of Diltiazem to Improve Coronary Vasomotor Dysfunction in ANOCA: The EDIT-CMD Randomized Clinical Trial. JACC Cardiovasc Imaging. 2022;15(8):1473-84.

S83. Group IS. Effect of nicorandil on coronary events in patients with stable angina: the Impact Of Nicorandil in Angina (IONA) randomised trial. Lancet. 2002;359(9314):1269-75.

S84. Yamabe H, Namura H, Yano T, Fujita H, Kim S, Iwahashi M, et al. Effect of nicorandil on abnormal coronary flow reserve assessed by exercise 201Tl scintigraphy in patients with angina pectoris and nearly normal coronary arteriograms. Cardiovasc Drugs Ther. 1995;9(6):755-61.

S85. Chen JW, Lee WL, Hsu NW, Lin SJ, Ting CT, Wang SP, et al. Effects of short-term treatment of nicorandil on exercise-induced myocardial ischemia and abnormal cardiac autonomic activity in microvascular angina. Am J Cardiol. 1997;80(1):32-8.

S86. Jia Q, Shi S, Yuan G, Shi J, Wei Y, Hu Y. The effect of nicorandil in patients with cardiac syndrome X: A meta-analysis of randomized controlled trials. Medicine (Baltimore). 2020;99(37):e22167.

S87. Lablanche JM, Bauters C, Leroy F, Bertrand ME. Prevention of coronary spasm by nicorandil: comparison with nifedipine. J Cardiovasc Pharmacol. 1992;20 Suppl 3:S82-5.

S88. Ya Li YL, Wanzhong Peng, Bingxun Wang, Tao Geng, Zesheng Xu. Therapeutic effect and safety of nicorandil in treatment of refractory angina pectoris. Int J Clin Exp Med. 2018;11(7):6993-8.

S89. Chaitman BR, Pepine CJ, Parker JO, Skopal J, Chumakova G, Kuch J, et al. Effects of ranolazine with atenolol, amlodipine, or diltiazem on exercise tolerance and angina frequency in patients with severe chronic angina: a randomized controlled trial. JAMA. 2004;291(3):309-16.

S90. Weisz G, Généreux P, Iñiguez A, Zurakowski A, Shechter M, Alexander KP, et al. Ranolazine in patients with incomplete revascularisation after percutaneous coronary intervention (RIVER-PCI): a multicentre, randomised, double-blind, placebo-controlled trial. Lancet. 2016;387(10014):136-45.

S91. Mehta PK, Goykhman P, Thomson LE, Shufelt C, Wei J, Yang Y, et al. Ranolazine improves angina in women with evidence of myocardial ischemia but no obstructive coronary artery disease. JACC Cardiovasc Imaging. 2011;4(5):514-22.

S92. Tagliamonte E, Rigo F, Cirillo T, Astarita C, Quaranta G, Marinelli U, et al. Effects of ranolazine on noninvasive coronary flow reserve in patients with myocardial ischemia but without obstructive coronary artery disease. Echocardiography. 2015;32(3):516-21.

S93. Bairey Merz CN, Handberg EM, Shufelt CL, Mehta PK, Minissian MB, Wei J, et al. A randomized, placebo-controlled trial of late Na current inhibition (ranolazine) in coronary microvascular dysfunction (CMD): impact on angina and myocardial perfusion reserve. Eur Heart J. 2016;37(19):1504-13.

S94. Villano A, Di Franco A, Nerla R, Sestito A, Tarzia P, Lamendola P, et al. Effects of ivabradine and ranolazine in patients with microvascular angina pectoris. Am J Cardiol. 2013;112(1):8-13.

S95. Koh JS, Hung OY, Eshtehardi P, Kumar A, Rabah R, Raad M, et al. Microvascular Assessment of Ranolazine in Non-Obstructive Atherosclerosis: The MARINA Randomized, Double-Blinded, Controlled Pilot Trial. Circ Cardiovasc Interv. 2020;13(12):e008204.

S96. Storey KM, Wang J, Garberich RF, Bennett NM, Traverse JH, Arndt TL, et al. Long-Term (3 Years) Outcomes of Ranolazine Therapy for Refractory Angina Pectoris (from the Ranolazine Refractory Registry). Am J Cardiol. 2020;129:1-4.

S97. Ling H, Packard KA, Burns TL, Hilleman DE. Impact of ranolazine on clinical outcomes and healthcare resource utilization in patients with refractory angina pectoris. Am J Cardiovasc Drugs. 2013;13(6):407-12.

S98. Ferrari R, Ford I, Fox K, Challeton JP, Correges A, Tendera M, et al. Efficacy and safety of trimetazidine after percutaneous coronary intervention (ATPCI): a randomised, double-blind, placebo-controlled trial. Lancet. 2020;396(10254):830-8.

S99. Ilic I, Timcic S, Milosevic M, Boskovic S, Odanovic N, Furtula M, et al. The imPAct of Trimetazidine on MicrOcirculation after Stenting for stable coronary artery disease (PATMOS study). Front Cardiovasc Med. 2023;10:1112198.

S100. Peng S, Zhao M, Wan J, Fang Q, Fang D, Li K. The efficacy of trimetazidine on stable angina pectoris: a meta-analysis of randomized clinical trials. Int J Cardiol. 2014;177(3):780-5.

S101. Nalbantgil S, Altinti&gbreve, A, Yilmaz H, Nalbantgil I, Önder R. The Effect of Trimetazidine in the Treatment of Microvascular Angina. Int J Angiol. 1999;8(1):40-3.

S102. Leonardo F, Fragasso G, Rossetti E, Dabrowski P, Pagnotta P, Rosano GM, et al. Comparison of trimetazidine with atenolol in patients with syndrome X: effects on diastolic function and exercise tolerance. Cardiologia. 1999;44(12):1065-9.

S103. Kim YH, Her AY, Rha SW, Choi BG, Choi SY, Byun JK, et al. Impact of Trimetazidine Treatment on 5-year Clinical Outcomes in Patients with Significant Coronary Artery Spasm: A Propensity Score Matching Study. Am J Cardiovasc Drugs. 2018;18(2):117-27.

S104. Ajabnoor A, Mukhtar A. Effect of trimetazidine on the functional capacity of ischemic heart disease patients not suitable for revascularization: Meta-analysis of randomized controlled trials. PLoS One. 2022;17(2):e0263932.

S105. Fox K, Ford I, Steg PG, Tendera M, Ferrari R, Investigators B. Ivabradine for patients with stable coronary artery disease and left-ventricular systolic dysfunction (BEAUTIFUL): a randomised, double-blind, placebo-controlled trial. Lancet. 2008;372(9641):807-16.

S106. Fox K, Ford I, Steg PG, Tardif JC, Tendera M, Ferrari R, et al. Ivabradine in stable coronary artery disease without clinical heart failure. N Engl J Med. 2014;371(12):1091-9.

S107. Zhu H, Xu X, Fang X, Zheng J, Zhao Q, Chen T, et al. Effects of the Antianginal Drugs Ranolazine, Nicorandil, and Ivabradine on Coronary Microvascular Function in Patients With Nonobstructive Coronary Artery Disease: A Meta-analysis of Randomized Controlled Trials. Clin Ther. 2019;41(10):2137-52.e12.

S108. Ceremuzyński L, Chamiec T, Herbaczyńska-Cedro K. Effect of supplemental oral L-arginine on exercise capacity in patients with stable angina pectoris. Am J Cardiol. 1997;80(3):331-3.

S109. Lerman A, Burnett JC, Higano ST, McKinley LJ, Holmes DR. Long-term L-arginine supplementation improves small-vessel coronary endothelial function in humans. Circulation. 1998;97(21):2123-8.

S110. Vicari RM, Chaitman B, Keefe D, Smith WB, Chrysant SG, Tonkon MJ, et al. Efficacy and safety of fasudil in patients with stable angina: a double-blind, placebo-controlled, phase 2 trial. J Am Coll Cardiol. 2005;46(10):1803-11.

S111. Suda A, Takahashi J, Hao K, Kikuchi Y, Shindo T, Ikeda S, et al. Coronary Functional Abnormalities in Patients With Angina and Nonobstructive Coronary Artery Disease. J Am Coll Cardiol. 2019;74(19):2350-60.

S112. Mohri M, Shimokawa H, Hirakawa Y, Masumoto A, Takeshita A. Rho-kinase inhibition with intracoronary fasudil prevents myocardial ischemia in patients with coronary microvascular spasm. J Am Coll Cardiol. 2003;41(1):15-9.

S113. Masumoto A, Mohri M, Shimokawa H, Urakami L, Usui M, Takeshita A. Suppression of coronary artery spasm by the Rho-kinase inhibitor fasudil in patients with vasospastic angina. Circulation. 2002;105(13):1545-7.

S114. Ford TJ, Stanley B, Good R, Rocchiccioli P, McEntegart M, Watkins S, et al. Stratified Medical Therapy Using Invasive Coronary Function Testing in Angina: The CorMicA Trial. J Am Coll Cardiol. 2018;72(23 Pt A):2841-55.

S115. Pauly DF, Johnson BD, Anderson RD, Handberg EM, Smith KM, Cooper-DeHoff RM, et al. In women with symptoms of cardiac ischemia, nonobstructive coronary arteries, and microvascular dysfunction, angiotensin-converting enzyme inhibition is associated with improved microvascular function: A double-blind randomized study from the National Heart, Lung and Blood Institute Women's Ischemia Syndrome Evaluation (WISE). Am Heart J. 2011;162(4):678-84.

S116. Masumoto A, Mohri M, Takeshita A. Three-year follow-up of the Japanese patients with microvascular angina attributable to coronary microvascular spasm. Int J Cardiol. 2001;81(2-3):151-6.

S117. Vervaat FE, van der Gaag A, Teeuwen K, van Suijlekom H, Wijnbergen I. Neuromodulation in patients with refractory angina pectoris: a review. Eur Heart J Open. 2023;3(1):oeac083.

S118. Henry TD, Satran D, Jolicoeur EM. Treatment of refractory angina in patients not suitable for revascularization. Nat Rev Cardiol. 2014;11(2):78-95.

S119. Parisi AF, Folland ED, Hartigan P. A comparison of angioplasty with medical therapy in the treatment of single-vessel coronary artery disease. Veterans Affairs ACME Investigators. N Engl J Med. 1992;326(1):10-6.

S120. Folland ED, Hartigan PM, Parisi AF. Percutaneous transluminal coronary angioplasty versus medical therapy for stable angina pectoris: outcomes for patients with double-vessel versus single-vessel coronary artery disease in a Veterans Affairs Cooperative randomized trial. Veterans Affairs ACME InvestigatorS. J Am Coll Cardiol. 1997;29(7):1505-11.

S121. Cameron AA, Davis KB, Rogers WJ. Recurrence of angina after coronary artery bypass surgery: predictors and prognosis (CASS Registry). Coronary Artery Surgery Study. J Am Coll Cardiol. 1995;26(4):895-9.

S122. Investigators S. Coronary artery bypass surgery versus percutaneous coronary intervention with stent implantation in patients with multivessel coronary artery disease (the Stent or Surgery trial): a randomised controlled trial. Lancet. 2002;360(9338):965-70.

S123. Taggart DP, Altman DG, Gray AM, Lees B, Nugara F, Yu LM, et al. Randomized trial to compare bilateral vs. single internal mammary coronary artery bypass grafting: 1-year results of the Arterial Revascularisation Trial (ART). Eur Heart J. 2010;31(20):2470-81.

S124. Hueb WA, Bellotti G, de Oliveira SA, Arie S, de Albuquerque CP, Jatene AD, et al. The Medicine, Angioplasty or Surgery Study (MASS): a prospective, randomized trial of medical therapy, balloon angioplasty or bypass surgery for single proximal left anterior descending artery stenoses. J Am Coll Cardiol. 1995;26(7):1600-5.

S125. Favarato ME, Hueb W, Boden WE, Lopes N, Nogueira CR, Takiuti M, et al. Quality of life in patients with symptomatic multivessel coronary artery disease: a comparative post hoc analyses of medical, angioplasty or surgical strategies-MASS II trial. Int J Cardiol. 2007;116(3):364-70.

S126. Serruys PW, Unger F, Sousa JE, Jatene A, Bonnier HJ, Schönberger JP, et al. Comparison of coronary-artery bypass surgery and stenting for the treatment of multivessel disease. N Engl J Med. 2001;344(15):1117-24.

S127. Coronary angioplasty versus medical therapy for angina: the second Randomised Intervention Treatment of Angina (RITA-2) trial. RITA-2 trial participants. Lancet. 1997;350(9076):461-8.

S128. Dagenais GR, Lu J, Faxon DP, Kent K, Lago RM, Lezama C, et al. Effects of optimal medical treatment with or without coronary revascularization on angina and subsequent revascularizations in patients with type 2 diabetes mellitus and stable ischemic heart disease. Circulation. 2011;123(14):1492-500.

S129. Boden WE, O'Rourke RA, Teo KK, Hartigan PM, Maron DJ, Kostuk WJ, et al. Optimal medical therapy with or without PCI for stable coronary disease. N Engl J Med. 2007;356(15):1503-16.

S130. Cohen DJ, Van Hout B, Serruys PW, Mohr FW, Macaya C, den Heijer P, et al. Quality of life after PCI with drug-eluting stents or coronary-artery bypass surgery. N Engl J Med. 2011;364(11):1016-26.

S131. Abdallah MS, Wang K, Magnuson EA, Spertus JA, Farkouh ME, Fuster V, et al. Quality of life after PCI vs CABG among patients with diabetes and multivessel coronary artery disease: a randomized clinical trial. JAMA. 2013;310(15):1581-90.

S132. Baron SJ, Chinnakondepalli K, Magnuson EA, Kandzari DE, Puskas JD, Ben-Yehuda O, et al. Quality-of-Life After Everolimus-Eluting Stents or Bypass Surgery for Left-Main Disease: Results From the EXCEL Trial. J Am Coll Cardiol. 2017;70(25):3113-22.

S133. Ono M, Serruys PW, Kawashima H, Lunardi M, Wang R, Hara H, et al. Impact of residual angina on long-term clinical outcomes after percutaneous coronary intervention or coronary artery bypass graft for complex coronary artery disease. Eur Heart J Qual Care Clin Outcomes. 2023;9(5):490-501.

S134. Spertus JA, Jones PG, Maron DJ, O'Brien SM, Reynolds HR, Rosenberg Y, et al. Health-Status Outcomes with Invasive or Conservative Care in Coronary Disease. N Engl J Med. 2020;382(15):1408-19.

S135. Singh A, Rodman J, Brown EL, Brown DL. Incidence, predictors and outcomes of angina-free status following revascularization for stable angina in the ISCHEMIA Trial. Journal of the American College of Cardiology. 2024;83(13_Supplement):1149-.

S136. Patel MR, Jeremias A, Maehara A, Matsumura M, Zhang Z, Schneider J, et al. 1-Year Outcomes of Blinded Physiological Assessment of Residual Ischemia After Successful PCI: DEFINE PCI Trial. JACC Cardiovasc Interv. 2022;15(1):52-61.

S137. Rajkumar CA, Foley MJ, Ahmed-Jushuf F, Nowbar AN, Simader FA, Davies JR, et al. A Placebo-Controlled Trial of Percutaneous Coronary Intervention for Stable Angina. N Engl J Med. 2023;389(25):2319-30.

S138. Mannheimer C, Camici P, Chester MR, Collins A, DeJongste M, Eliasson T, et al. The problem of chronic refractory angina; report from the ESC Joint Study Group on the Treatment of Refractory Angina. Eur Heart J. 2002;23(5):355-70.

S139. McGillion M, Arthur HM, Cook A, Carroll SL, Victor JC, L'allier PL, et al. Management of patients with refractory angina: Canadian Cardiovascular Society/Canadian Pain Society joint guidelines. Can J Cardiol. 2012;28(2 Suppl):S20-41.

S140. Gulati M, Levy PD, Mukherjee D, Amsterdam E, Bhatt DL, Birtcher KK, et al. 2021 AHA/ACC/ASE/CHEST/SAEM/SCCT/SCMR Guideline for the Evaluation and Diagnosis of Chest Pain: A Report of the American College of Cardiology/American Heart Association Joint Committee on Clinical Practice Guidelines. Circulation. 2021;144(22):e368-e454.

S141. Messin R, Opolski G, Fenyvesi T, Carreer-Bruhwyler F, Dubois C, Famaey JP, et al. Efficacy and safety of molsidomine once-a-day in patients with stable angina pectoris. Int J Cardiol. 2005;98(1):79-89.

S142. Danchin N, Juillière Y, Anconina J, Perrin O, Selton-Suty C, Cherrier F. Comparative effects of oral molsidomine and nifedipine on methylergometrine-induced coronary artery spasm. Am J Cardiol. 1991;67(15):1208-11.

S143. Horgan JH, O'Callaghan WG, Teo KK. Therapy of angina pectoris with low-dose perhexiline. J Cardiovasc Pharmacol. 1981;3(3):566-72.

S144. Waters DD, Miller DD, Szlachcic J, Bouchard A, Méthé M, Kreeft J, et al. Factors influencing the long-term prognosis of treated patients with variant angina. Circulation. 1983;68(2):258-65.

S145. Cole PL, Beamer AD, McGowan N, Cantillon CO, Benfell K, Kelly RA, et al. Efficacy and safety of perhexiline maleate in refractory angina. A double-blind placebo-controlled clinical trial of a novel antianginal agent. Circulation. 1990;81(4):1260-70.

S146. Phan TT, Shivu GN, Choudhury A, Abozguia K, Davies C, Naidoo U, et al. Multi-centre experience on the use of perhexiline in chronic heart failure and refractory angina: old drug, new hope. Eur J Heart Fail. 2009;11(9):881-6.

S147. Noman A, Ang DS, Ogston S, Lang CC, Struthers AD. Effect of high-dose allopurinol on exercise in patients with chronic stable angina: a randomised, placebo controlled crossover trial. Lancet. 2010;375(9732):2161-7.

S148. Lim TK, Noman A, Choy AMJ, Khan F, Struthers AD, Lang CC. The APEX trial: Effects of allopurinol on exercise capacity, coronary and peripheral endothelial function, and natriuretic peptides in patients with cardiac syndrome X. Cardiovasc Ther. 2018;36(1).

S149. Dzerve V, Group MIS. A dose-dependent improvement in exercise tolerance in patients with stable angina treated with mildronate: a clinical trial "MILSS I". Medicina (Kaunas). 2011;47(10):544-51.

S150. LaRosa JC, Grundy SM, Waters DD, Shear C, Barter P, Fruchart JC, et al. Intensive lipid lowering with atorvastatin in patients with stable coronary disease. N Engl J Med. 2005;352(14):1425-35.

S151. Cannon CP, Braunwald E, McCabe CH, Rader DJ, Rouleau JL, Belder R, et al. Intensive versus moderate lipid lowering with statins after acute coronary syndromes. N Engl J Med. 2004;350(15):1495-504.

S152. Randomised trial of cholesterol lowering in 4444 patients with coronary heart disease: the Scandinavian Simvastatin Survival Study (4S). Lancet. 1994;344(8934):1383-9.

S153. Manfrini O, Amaduzzi P, Bergami M, Cenko E. Effects of Statin Treatment on Patients with Angina and Normal or Nearly Normal Angiograms. Eur Cardiol. 2020;15:e15.

S154. Yasue H, Mizuno Y, Harada E, Itoh T, Nakagawa H, Nakayama M, et al. Effects of a 3-hydroxy-3-methylglutaryl coenzyme A reductase inhibitor, fluvastatin, on coronary spasm after withdrawal of calcium-channel blockers. J Am Coll Cardiol. 2008;51(18):1742-8.

S155. Suhrs HE, Nilsson M, Bové KB, Zander M, Prescott E. Effect of empagliflozin on coronary microvascular function in patients with type 2 diabetes mellitus-A randomized, placebo-controlled cross-over study. PLoS One. 2022;17(2):e0263481.

S156. Mansouri MH, Mansouri P, Sadeghi M, Hashemi SM, Khosravi A, Behjati M, et al. Antianginal effects of empagliflozin in patients with type 2 diabetes and refractory angina; a randomized, double-blind placebo-controlled trial (EMPT-ANGINA Trial). Clin Cardiol. 2024;47(1):e24158.

S157. Lin JL, Tseng WK, Lee PT, Lee CH, Tseng SY, Chen PW, et al. A Randomized Controlled Trial Evaluating Outcome Impact of Cilostazol in Patients with Coronary Artery Disease or at a High Risk of Cardiovascular Disease. J Pers Med. 2022;12(6).

S158. Watanabe K, Ikeda S, Komatsu J, Inaba S, Suzuki J, Sueda S, et al. Effect of cilostazol on vasomotor reactivity in patients with vasospastic angina pectoris. Am J Cardiol. 2003;92(1):21-5.

S159. Shin ES, Lee JH, Yoo SY, Park Y, Hong YJ, Kim MH, et al. A randomised, multicentre, double blind, placebo controlled trial to evaluate the efficacy and safety of cilostazol in patients with vasospastic angina. Heart. 2014;100(19):1531-6.

S160. Kang MG, Ahn JH, Hwang JY, Hwang SJ, Koh JS, Park Y, et al. Long-acting cilostazol versus isosorbide mononitrate for patients with vasospastic angina: a randomized controlled trial. Coron Artery Dis. 2024;35(6):459-64.

S161. Fox KM, Thadani U, Ma PT, Nash SD, Keating Z, Czorniak MA, et al. Sildenafil citrate does not reduce exercise tolerance in men with erectile dysfunction and chronic stable angina. Eur Heart J. 2003;24(24):2206-12.

S162. Denardo SJ, Wen X, Handberg EM, Bairey Merz CN, Sopko GS, Cooper-Dehoff RM, et al. Effect of phosphodiesterase type 5 inhibition on microvascular coronary dysfunction in women: a Women's Ischemia Syndrome Evaluation (WISE) ancillary study. Clin Cardiol. 2011;34(8):483-7.

S163. Morrow A, Young R, Abraham GR, Hoole S, Greenwood JP, Arnold JR, et al. Zibotentan in Microvascular Angina: A Randomized, Placebo-Controlled, Crossover Trial. Circulation. 2024.

S164. Feenstra RGT, Jansen TPJ, Matthijs Boekholdt S, Brouwer JE, Klees MI, Appelman Y, et al. Efficacy and safety of the endothelin-1 receptor antagonist macitentan in epicardial and microvascular vasospasm; a proof-of-concept study. Int J Cardiol Heart Vasc. 2023;47:101238.

S165. Tebaldi M, Campo G, Ugo F, Guarracini S, Marrone A, Clò S, et al. Coronary Sinus Narrowing Improves Coronary Microcirculation Function in Patients With Refractory Angina: A Multicenter Prospective INROAD Study. Circ Cardiovasc Interv. 2024;17(1):e013481.

S166. Giannini F, Palmisano A, Baldetti L, Benedetti G, Ponticelli F, Rancoita PMV, et al. Patterns of Regional Myocardial Perfusion Following Coronary Sinus Reducer Implantation: Insights by Stress Cardiac Magnetic Resonance. Circ Cardiovasc Imaging. 2019;12(9):e009148.

S167. Palmisano A, Giannini F, Rancoita P, Gallone G, Benedetti G, Baldetti L, et al. Feature tracking and mapping analysis of myocardial response to improved perfusion reserve in patients with refractory angina treated by coronary sinus Reducer implantation: a CMR study. Int J Cardiovasc Imaging. 2021;37(1):291-303.

S168. Tzanis G, Palmisano A, Gallone G, Ponticelli F, Baldetti L, Esposito A, et al. The impact of the coronary sinus reducer upon left ventricular function in patients with refractory angina pectoris. Catheter Cardiovasc Interv. 2020;95(6):1104-8.

S169. Cheng K, Tan ST, Wechalekar K, Keramida G, de Silva R. Redistribution of myocardial perfusion after coronary sinus reducer implantation demonstrated by rubidium-82 positron emission tomography. J Nucl Cardiol. 2024;33:101803.

S170. Cheng K, Alpendurada F, Falaschetti E, Pennell D, Bucciarelli-Ducci C, De Silva R. Changes in myocardial perfusion after coronary sinus reducer implantation for refractory angina - assessment using fully automated quantitative stress perfusion cardiac MRI. European Heart Journal2023. p. ehad655.1294.

S171. Foley MJ, Rajkumar CA, Ahmed-Jushuf F, Simader FA, Chotai S, Pathimagaraj RH, et al. Coronary sinus reducer for the treatment of refractory angina (ORBITA-COSMIC): a randomised, placebo-controlled trial. Lancet. 2024;403(10436):1543-53.

S172. Verheye S, Jolicœur EM, Behan MW, Pettersson T, Sainsbury P, Hill J, et al. Efficacy of a device to narrow the coronary sinus in refractory angina. N Engl J Med. 2015;372(6):519-27.

S173. Arora RR, Chou TM, Jain D, Fleishman B, Crawford L, McKiernan T, et al. The multicenter study of enhanced external counterpulsation (MUST-EECP): effect of EECP on exercise-induced myocardial ischemia and anginal episodes. J Am Coll Cardiol. 1999;33(7):1833-40.

S174. Henry TD, Losordo DW, Traverse JH, Schatz RA, Jolicoeur EM, Schaer GL, et al. Autologous CD34+ cell therapy improves exercise capacity, angina frequency and reduces mortality in no-option refractory angina: a patient-level pooled analysis of randomized double-blinded trials. Eur Heart J. 2018;39(23):2208-16.

S175. Wojakowski W, Jadczyk T, Michalewska-Włudarczyk A, Parma Z, Markiewicz M, Rychlik W, et al. Effects of Transendocardial Delivery of Bone Marrow-Derived CD133. Circ Res. 2017;120(4):670-80.

S176. Jones DA, Weeraman D, Colicchia M, Hussain MA, Veerapen D, Andiapen M, et al. The Impact of Cell Therapy on Cardiovascular Outcomes in Patients With Refractory Angina. Circ Res. 2019;124(12):1786-95.

S177. Velagapudi P, Turagam M, Kolte D, Khera S, Hyder O, Gordon P, et al. Intramyocardial autologous CD34+ cell therapy for refractory angina: A meta-analysis of randomized controlled trials. Cardiovasc Revasc Med. 2019;20(3):215-9.

S178. Henry TD, Bairey Merz CN, Wei J, Corban MT, Quesada O, Joung S, et al. Autologous CD34+ Stem Cell Therapy Increases Coronary Flow Reserve and Reduces Angina in Patients With Coronary Microvascular Dysfunction. Circ Cardiovasc Interv. 2022;15(2):e010802.

S179. Corban MT, Toya T, Albers D, Sebaali F, Lewis BR, Bois J, et al. IMPROvE-CED Trial: Intracoronary Autologous CD34+ Cell Therapy for Treatment of Coronary Endothelial Dysfunction in Patients With Angina and Nonobstructive Coronary Arteries. Circ Res. 2022;130(3):326-38.

S180. Kastrup J, Jørgensen E, Rück A, Tägil K, Glogar D, Ruzyllo W, et al. Direct intramyocardial plasmid vascular endothelial growth factor-A165 gene therapy in patients with stable severe angina pectoris A randomized double-blind placebo-controlled study: the Euroinject One trial. J Am Coll Cardiol. 2005;45(7):982-8.

S181. Henry TD, Grines CL, Watkins MW, Dib N, Barbeau G, Moreadith R, et al. Effects of Ad5FGF-4 in patients with angina: an analysis of pooled data from the AGENT-3 and AGENT-4 trials. J Am Coll Cardiol. 2007;50(11):1038-46.

S182. Hartikainen J, Hassinen I, Hedman A, Kivelä A, Saraste A, Knuuti J, et al. Adenoviral intramyocardial VEGF-DΔNΔC gene transfer increases myocardial perfusion reserve in refractory angina patients: a phase I/IIa study with 1-year follow-up. Eur Heart J. 2017;38(33):2547-55.

S183. Nakamura K, Henry TD, Traverse JH, Latter DA, Mokadam NA, Answini GA, et al. Angiogenic Gene Therapy for Refractory Angina: Results of the EXACT Phase 2 Trial. Circ Cardiovasc Interv. 2024;17(5):e014054.

S184. Kikuchi Y, Ito K, Ito Y, Shiroto T, Tsuburaya R, Aizawa K, et al. Double-blind and placebo-controlled study of the effectiveness and safety of extracorporeal cardiac shock wave therapy for severe angina pectoris. Circ J. 2010;74(3):589-91.

S185. Schmid JP, Capoferri M, Wahl A, Eshtehardi P, Hess OM. Cardiac shock wave therapy for chronic refractory angina pectoris. A prospective placebo-controlled randomized trial. Cardiovasc Ther. 2013;31(3):e1-6.

S186. Yang P, Guo T, Wang W, Peng YZ, Wang Y, Zhou P, et al. Randomized and double-blind controlled clinical trial of extracorporeal cardiac shock wave therapy for coronary heart disease. Heart Vessels. 2013;28(3):284-91.

S187. Shindo T, Ito K, Ogata T, Kurosawa R, Eguchi K, Kagaya Y, et al. A randomized, double-blind, placebo-controlled pilot trial of low-intensity pulsed ultrasound therapy for refractory angina pectoris. PLoS One. 2023;18(6):e0287714.

S188. Cannon RO, Quyyumi AA, Mincemoyer R, Stine AM, Gracely RH, Smith WB, et al. Imipramine in patients with chest pain despite normal coronary angiograms. N Engl J Med. 1994;330(20):1411-7.

S189. Cox ID, Hann CM, Kaski JC. Low dose imipramine improves chest pain but not quality of life in patients with angina and normal coronary angiograms. Eur Heart J. 1998;19(2):250-4.

S190. Jiang W, Velazquez EJ, Kuchibhatla M, Samad Z, Boyle SH, Kuhn C, et al. Effect of escitalopram on mental stress-induced myocardial ischemia: results of the REMIT trial. JAMA. 2013;309(20):2139-49.

S191. Nitz J, Cheras F. Transcutaneous electrical nerve stimulation and chronic intractable angina pectoris. Aust J Physiother. 1993;39(2):109-13.

S192. West PD, Colquhoun DM. TENS in refractory angina pectoris. Three case reports. Med J Aust. 1993;158(7):488-9.

S193. Meyler WJ, de Jongste MJ, Rolf CA. Clinical evaluation of pain treatment with electrostimulation: a study on TENS in patients with different pain syndromes. Clin J Pain. 1994;10(1):22-7.

S194. Buiten MS, DeJongste MJ, Beese U, Kliphuis C, Durenkamp A, Staal MJ. Subcutaneous electrical nerve stimulation: a feasible and new method for the treatment of patients with refractory angina. Neuromodulation. 2011;14(3):258-65; discussion 65.

S195. Goroszeniuk T, Pang D, Al-Kaisy A, Sanderson K. Subcutaneous target stimulation-peripheral subcutaneous field stimulation in the treatment of refractory angina: preliminary case reports. Pain Pract. 2012;12(1):71-9.

S196. Eddicks S, Maier-Hauff K, Schenk M, Müller A, Baumann G, Theres H. Thoracic spinal cord stimulation improves functional status and relieves symptoms in patients with refractory angina pectoris: the first placebo-controlled randomised study. Heart. 2007;93(5):585-90.

S197. Lanza GA, Grimaldi R, Greco S, Ghio S, Sarullo F, Zuin G, et al. Spinal cord stimulation for the treatment of refractory angina pectoris: a multicenter randomized single-blind study (the SCS-ITA trial). Pain. 2011;152(1):45-52.

S198. Zipes DP, Svorkdal N, Berman D, Boortz-Marx R, Henry T, Lerman A, et al. Spinal cord stimulation therapy for patients with refractory angina who are not candidates for revascularization. Neuromodulation. 2012;15(6):550-8; discussion 8-9.

S199. Eldabe S, Thomson S, Duarte R, Brookes M, deBelder M, Raphael J, et al. The Effectiveness and Cost-Effectiveness of Spinal Cord Stimulation for Refractory Angina (RASCAL Study): A Pilot Randomized Controlled Trial. Neuromodulation. 2016;19(1):60-70.

S200. Denby C, Groves DG, Eleuteri A, Tsang HK, Leach A, Hammond C, et al. Temporary sympathectomy in chronic refractory angina: a randomised, double-blind, placebo-controlled trial. Br J Pain. 2015;9(3):142-8.

S201. Asbury EA, Webb CM, Probert H, Wright C, Barbir M, Fox K, et al. Cardiac rehabilitation to improve physical functioning in refractory angina: a pilot study. Cardiology. 2012;122(3):170-7.

S202. Mittal TK, Evans E, Pottle A, Lambropoulos C, Morris C, Surawy C, et al. Mindfulness-based intervention in patients with persistent pain in chest (MIPIC) of non-cardiac cause: a feasibility randomised control study. Open Heart. 2022;9(1).

S203. Moore RK, Groves D, Bateson S, Barlow P, Hammond C, Leach AA, et al. Health related quality of life of patients with refractory angina before and one year after enrolment onto a refractory angina program. Eur J Pain. 2005;9(3):305-10.

S204. Moore RK, Groves DG, Bridson JD, Grayson AD, Wong H, Leach A, et al. A brief cognitive-behavioral intervention reduces hospital admissions in refractory angina patients. J Pain Symptom Manage. 2007;33(3):310-6.

S205. Patel PA, Khan M, Yay C, Thapar S, Taylor S, Sainsbury PA. The short- and long-term impact of psychotherapy in patients with chronic, refractory angina. Br J Cardiol. 2016;23:57-60.

S206. Tinson D, Swartzman S, Lang K, Spense S, Todd I. Clinical and psychological outcomes of an angina management programme. Br J Cardiol. 2016;23:61-4.
